# Hydrazone Molecular Switches with Paramagnetic Center as ^19^F Magnetic Resonance Imaging Relaxation Enhancement Agents for pH Imaging

**DOI:** 10.1021/acssensors.3c00080

**Published:** 2023-05-18

**Authors:** Dawid Janasik, Krzysztof Jasiński, Julia Szreder, Władysław P. Węglarz, Tomasz Krawczyk

**Affiliations:** † Department of Chemical Organic Technology and Petrochemistry, Silesian University of Technology, 44-100 Gliwice, Poland; ‡ Institute of Nuclear Physics Polish Academy of Sciences, 31-342 Krakow, Poland

**Keywords:** molecular switches, ^19^F magnetic resonance imaging, pH, hydrazone, paramagnetic relaxation enhancement

## Abstract

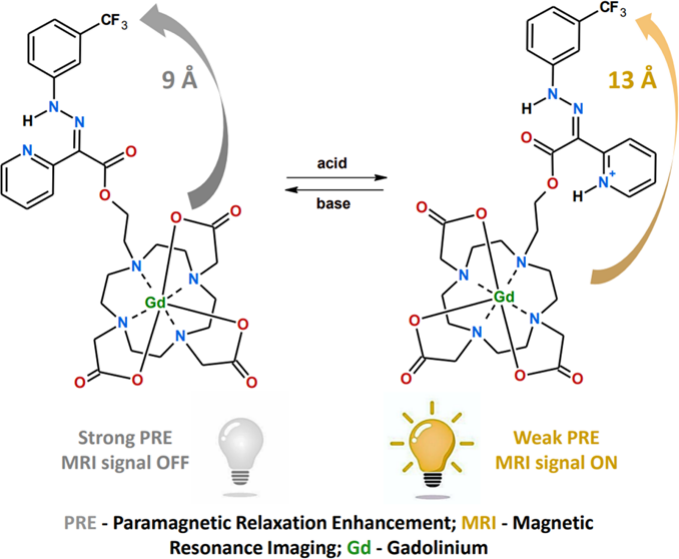

The design and synthesis of hydrazone-based switches with a CF_3_ reporting group for ^19^F pH imaging using relaxation rate changes were described. A paramagnetic center was introduced into the hydrazone molecular switch scaffold by substitution of an ethyl functional group with a paramagnetic complex. The mechanism of activation relies on a gradual increase in *T*_1_ and *T*_2_ magnetic resonance imaging (MRI) relaxation times as pH decreases due to *E*/*Z* isomerization, which results in a change in the distance between fluorine atoms and the paramagnetic center. Among the three possible variants of the ligand, the meta isomer was found to offer the highest potential changes in relaxation rates due to the significant paramagnetic relaxation enhancement (PRE) effect and a stable position of the ^19^F signal, allowing for the tracking of a single narrow ^19^F resonance for imaging purposes. The selection of the most suitable Gd(III) paramagnetic ion for complexation was conducted by theoretical calculations based on the Bloch–Redfield–Wangsness (BRW) theory, taking into account the electron–nucleus dipole–dipole and Curie interactions only. The results were verified experimentally, confirming the accuracy of theoretical predictions, good solubility, and stability of the agents in water and the reversible transition between E and Z–H^+^ isomers. The results demonstrate the potential of this approach for pH imaging using relaxation rate changes instead of chemical shift.

In recent years, molecular switches have gained significant importance in a variety of fields, including sensor technology, data and energy storage, drug delivery, and molecular machines.^1−3^ This is due to their ability to undergo reversible transitions between two or more states under the influence of external stimuli.^4,5^ Molecular switches can be activated by a range of stimuli, including light,^6,7^ chemicals,^8,9^ electricity,^10,11^ temperature,^12^ and electron tunneling.^2,13^ There are various mechanisms of switching, including conformation, bond, spin, dipole, and charge switching, which determine the physical or chemical property that allows for the observation of the switching process.^2,3,14^ Color-changing switches are the most common,^15,16^ but changes in electrical properties, such as potential or conductivity,^17,18^ or magnetic and spin properties, which are useful in nuclear magnetic resonance (NMR) imaging techniques, are also possible.^9,19^ In the latter case, these switches can be applied as contrast agents in either ^1^H or ^19^F magnetic resonance imaging (MRI).^9^

MRI is a noninvasive diagnostic technique of soft tissues with a superb spatial resolution that typically utilizes the magnetic properties of the ^1^H nucleus.^20^ It is one of the most important imaging techniques commonly used in hospitals and provides detailed information on soft tissues.^21,22^ Recently, there has been a growing interest in the potential use of ^19^F MRI as an additional modality to ^1^H MRI. Because the ^19^F atoms are not present in soft tissues, the modality offers efficient visualization of the targeted organ without any confounding endogenous background signal.^23^ Other attractive properties of the fluorine nucleus include its nonquadrupolar nature, a wide range of chemical shifts, a high gyromagnetic ratio, 100% natural abundance, and similar sensitivity to ^1^H. Additionally, it is possible to use existing clinical MR scanners for both ^1^H and ^19^F modalities after slight hardware modifications.^24^

Fluorine-based MRI is well-suited for visualizing bioactive molecules or biological parameters in vivo.^25,26^ It can be an important tool in the more personalized medicinal diagnosis and for understanding and rationalizing the molecular factors underlying physiological and pathological processes.^27^ For this purpose, responsive molecular MRI probes can be designed to report on various biomarkers of biological interest.^9,28^ So far, several smart ^19^F MRI contrast agents have been proposed, including pH-,^29−33^ redox-,^34−37^ Ca^2+^-,^38,39^ and enzyme^40,41^-responsive agents. Among different types of stimuli, pH gradients have been frequently used as a trigger for environmentally responsive tumor imaging.^42^

The principle of molecular imaging with ^19^F MRI can rely on variations in chemical shift or relaxation properties of fluorine atoms of a contrast agent. The former has been reported in the case of redox-sensitive^37,43^ agents or agents with varying hydration numbers.^38^ Changes in relaxation rates can be observed in nanoparticle-based agents where encapsulating the fluorine carrier in a capsule or network decreases the mobility of ^19^F nuclei, resulting in the increased intensity of the NMR signal.^44−47^ Another approach uses the paramagnetic resonance enhancement (PRE) effect to modulate relaxation times and requires the presence of a paramagnetic center. In this case, the external stimuli alter the distance between fluorine atoms and the paramagnet. This approach commonly uses linkers that break under the influence of low pH or the presence of an enzyme, resulting in a reduced PRE effect and increased signal intensity.^29,31,48,49^

The aim of this study is to propose a fully reversible pH-sensitive ^19^F MRI contrast agent based on a fluorinated hydrazone molecular switch containing a pendant paramagnetic complex. The pH sensitivity of the agent is achieved by an increase in the distance of the paramagnetic center from the CF_3_ group resulting from *E*/*Z* isomerization of the switch induced by a pH 5–4. Change in the distance modulates the paramagnetic resonance enhancement (PRE) effect, leading to lengthening of *T*_1_ and *T*_2_ relaxation times of fluorine nuclei but no changes in the chemical shift of the CF_3_ reporter group. This approach is a significant improvement over previous pH-sensitive agents, which were not reversible and had limited practical applications.

## Experimental Section

Unless otherwise noted, all reagents and starting materials were purchased from commercial vendors and used without further purification. All experiments were conducted under air unless otherwise noted. Column chromatography was performed using silica gel (60 Å, 230–400 mesh).

### Syntheses

#### Ethyl-2-(pyridin-2-yl)acetate (**1**)

*n*-BuLi (2.5 M solution in hexanes, 26.4 mL, 66.0 mmol, 2.05 equiv) was added dropwise to a stirred solution of diisopropylamine (6.8 g, 9.5 mL, 68 mmol, 2.1 equiv) in tetrahydrofuran (THF) (40 mL) at −78 °C under argon (Ar). The resulting solution was warmed in 1 h to 0 °C and stirred at 0 °C for another 1 h. Then, the solution was transferred to a stirred solution of 2-picoline (3.0 g, 3.2 mL, 32.0 mmol, 1.0 equiv) and diethyl carbonate (11.4 g, 11.7 mL, 97 mmol, 3.0 equiv) in THF (40 mL) at −78 °C under Ar. The resulting solution was stirred at −78 °C for 1 h and then allowed to warm in 1 h to room temperature (rt) and stirred for 30 min. Saturated NH_4_Cl (aq) (20 mL) and water (50 mL) were added, the two layers (organic and aqueous) were separated, and the aqueous layer was extracted with Et_2_O (3 × 30 mL). The combined organic layers were dried with 7 g of MgSO_4_ and evaporated under reduced pressure to give the product (4.5 g, 85%) as a bright yellow oil.^9^

^1^H NMR (400 MHz, CDCl_3_, 298 K) δ = 8.55 (d, *J* = 7.0 Hz 1H), 7.66 (t, *J* = 7.0 Hz, 1H), 7.29 (d, *J* = 7.0 Hz, 1H), 7.19 (dd, *J* = 7.0, 5,0 Hz, 1H), 4,18 (q, *J* = 7.0 Hz, 2H), 3,84 (s, 2H), 1.26 (t, *J* = 7.0 Hz, 3H); ^13^C NMR (100 MHz, CDCl_3_) δ = 170.7, 154.5, 149.5, 136.7, 123.9, 122.1, 61.1, 40.0, 14.2; HR-MS (ESI): *m*/*z* calcd for C_9_H_12_NO_2_, [M – H]^+^, 166.0868; found: 166.0865.

#### Ethyl-(2*E*)-(pyridin-2-yl)-{2-[3-(trifluoromethyl)phenyl]hydrazinylidene}acetate (**2**^**b**^)

Trifluoromethylaniline (1.0 g, 6.2 mmol, 1.0 equiv) was dissolved in a mixture of 10.0 mL of 36% HCl and 10.0 mL of 99% EtOH and stirred in an ice bath for 30 min. Solution (8.0 mL) of sodium nitrite (0.4 g, 6.2 mmol, 1.0 equiv) was then added dropwise to the acidified solution over a period of 30 min. The obtained diazonium salt solution was then added dropwise to a suspension of ethyl-2-pyridylacetate (1.0 mL, 6.2 mmol, 1.0 equiv) and sodium acetate (3.3 g, 36.7 mmol, 6.4 equiv) in a cooled 40 mL EtOH/water (8:1) mixture. The resulting reaction mixture was stirred overnight and then washed with dichloromethane (DCM). The organic fraction was washed twice with 30 mL of saturated NaHCO_3_ solution and dried over MgSO_4_. The crude product was then subjected to silica gel column chromatography (methanol/methylene chloride 1:8) to give the pure compound as a bright orange solid (1.3 g, 63%).^9^

^1^H NMR (400 MHz, CDCl_3_, 298 K) δ = 14.89 (s, 1H), 8.66 (ddd, *J* = 5.0, 2.0, 0.5 Hz, 1H), 8.23 (dt, *J* = 8.0, 2.0 Hz, 1H), 7.83 (td, *J* = 7.5, 1.5 Hz, 1H), 7.58 (s, 1H), 7.54 (d, *J* = 8.0 Hz, 1H), 7.44 (t, *J* = 8.0 Hz, 1H), 7.30 (ddd, *J* = 7.9, 2.1, 0.5 Hz, 1H), 7.25 (d, *J* = 7.9 Hz, 1H), 4.40 (q, *J* = 7.1 Hz, 2H), 1.45 (t, *J* = 7.1 Hz, 3H); ^13^C NMR (100 MHz, CDCl_3_) δ = 165.4, 152.4 S5, 146.5, 143.9, 136.9, 131.5 (q, *J* = 3.1 Hz), 129.8, 126.9, 124.5, 123.4, 122.7, 118.9, 117.7, 111.5, 61.2, 14.3; HR-MS (ESI): *m*/*z* calcd for C_16_H_15_N_3_O_2_F_3_, [M – H]^+^, 338.1116; found 338.1119.

#### (2*E*)-(Pyridin-2-yl){2-[3-(trifluoromethyl)phenyl]hydrazinylidene}acetic Acid (**3**^**b**^)

10 mL of MeOH was heated to 35 °C and (2) was dissolved (1.3 g, 3.8 mmol, 1 equiv) with KOH (0.6 g, 11.4 mmol, 3 equiv). The mixture was stirred for 2 h and then quenched with 5 mL of water. The aqueous layer was washed with Et_2_O (3 × 5 mL), and the inorganic phase was acidified with HCl to obtain pH = 3. The resulting precipitate was filtered and dried to obtain the product as a fluffy yellow powder (0.9 g, 82%).

^1^H NMR (400 MHz, CDCl_3_, 298 K) δ = 13.99 (s, 1H), 8.39 (ddd, *J* = 5.0, 2.0, 0.5 Hz, 1H), 8.23 (dt, *J* = 8.0, 2.0 Hz, 1H), 7.94 (td, *J* = 7.5, 1.5 Hz, 1H), 7.68 (s, 1H), 7.50 (m, 2H), 7.35 (m, 1H), 1.72 (s, OH); ^13^C NMR (100 MHz, CDCl_3_) δ = 167.1, 154.2, 143.0, 142.8, 132.3, 130.1, 128.0, 125.3, 122.4, 120.4, 120.2, 118.4, 111.7. HR-MS (ESI): *m*/*z* calcd for C_14_H_11_N_3_O_2_F_3_, [M – H]^+^, 310.0803; found 310.0814.

#### *tert*-Butyl-2,2′,2″-(1,4,7,10-tetraazacyclododecane-1,4,7- triyl)triacetate (**4**)

40 mL of anhydrous acetonitrile (ACN), NaHCO_3_ (2.77 g, 33 mmol, 3.3 equiv), and 1,4,7,10-tetraazacyclododecane (1.72 g, 10.0 mmol, 1.0 equiv) were mixed in an ice bath under Ar. Then, *tert*-butyl bromoacetate (4.81 mL, 33 mmol, 3.3 equiv) was slowly added dropwise. The reaction was carried out for 48 h at room temperature and monitored by ultraperformance liquid chromatography-mass spectrometry (UPLC-MS). After completion of the reaction, the mixture was filtered, and the solvent was evaporated. The resulting yellow-brown precipitate was recrystallized several times in hot toluene until a white solid was obtained (3.0 g, 45%).

^1^H NMR (400 MHz, CDCl_3_): δ 10.00 (s, 1H), 3.38 (s, 4H), 3.30 (s, 2H), 3.14 (m, 4H), 2.83 (m, 10H), 1.76 (s, 2H), 1.49 (m, 27H); ^13^C NMR (100 MHz, CDCl_3_): δ 172.9, 170.5, 169.6, 81.80 (2C), 81.6, 58.2(2C), 55.7, 54.3, 51.3, 51.2, 50.5, 49.2, 48.9 (2C), 47.5 (2C), 28.1 (5C), 28.0, 27.9 (3C). HR-MS (ESI): *m*/*z* calcd for C_26_H_51_N_4_O_6_, [M – H]^+^, 515.3809; found 515.3801.

#### *tert*-Butyl 2,2′,2″-(10-(2-Hydroxyethyl)-1,4,7,10-tetraazacyclododecane-1,4,7-triyl)triacetate (**5**)

To a mixture of anhydrous ACN (20 mL) and anhydrous K_2_CO_3_ (0.6 g, 4.5 mmol, 5 equiv) under an atmosphere of N_2_ was added compound (**4**) (0.5 g, 1.0 mmol, 1 equiv) followed by 2-bromoethanol (320 μL, 4.5 mmol, 5 equiv). The resulting mixture was stirred at rt for 7 days. Excess K_2_CO_3_ was removed by filtration, and the solvent was removed under reduced pressure. The resulting solid was washed 3 times with hot toluene. The filtrate was condensed under reduced pressure and purified using silica gel chromatography (10:1 DCM/MeOH) to give (**5**) as a light green/yellow oil (0.5 g, 90%).

^1^H NMR (400 MHz, CDCl_3_, 298 K) δ = 3.52 (s, 2H), 3.27 (m, 6H), 2.83 (m, 7H), 2.77 (m, 4H), 2.54 (m, 5H), 2.34 (s, 1H), 2.10 (s, 2H), 1.44 (m, 27H); ^13^C NMR (100 MHz, CDCl_3_) δ = 171.2, 171.0, 170.9, 155.3, 137.8, 129.0, 128.2, 125.2, 80.8, 80.7, 77.2, 64.5, 59.7, 59.6, 56.7, 56.2, 56.1, 55.9, 53.5, 52.5, 52.1, 52.0, 50.8, 47.4, 28.2, 28.0, 27.9, 21.4. HR-MS (ESI): *m*/*z* calcd for C_28_H_55_N_4_O_7_, [M – H]^+^, 559.4070; found 559.4063.

#### 10-Ethyl-[*tert*-butyl-2,2′,2″-(1,4,7,10-tetraazacyclododecane-1,4,7-triyl)triacetate]-(2*E*)-(pyridin-2-yl)-{2-[3-(trifluoromethyl)phenyl]hydrazinylidene}acetate (**6**^**b**^)

Compounds (**3**^**b**^) (0.2 g, 0.6 mmol, 1 equiv) and (**5**) (0.5 g, 1 mmol, 1.7 equiv) were dissolved in 10 mL of anhydrous DCM with DCC (0.4 g, 1.8 mmol, 3 equiv) and 4-dimethylaminopyridine (DMAP) (cat.). The reaction was stirred under Ar at rt for 14 days. Then, the reaction was filtered and condensed under reduced pressure. The crude product was purified using silica gel chromatography (10:2 DCM/MeOH) to give (**6**^**b**^) as a light orange powder (0.4 g, 84%).

^1^H NMR (400 MHz, CDCl_3_, 298 K) δ = 15.13 (s, 1H), 8.65 (ddd, *J* = 5.0, 2.0, 0.5 Hz, 1H), 8.28 (dt, *J* = 8.0, 2.0 Hz, 1H), 7.83 (td, *J* = 7.5, 1.5 Hz, 1H), 7.51 (s, 1H), 7.54 (d, *J* = 8.0 Hz, 1H), 7.44 (m, 2H), 7.32 (m, 2H), 4.45 (t, *J* = 7.0 Hz, 2H), 3.04 (m, 6H), 2.85 (t, *J* = 7.1 Hz, 2H), 1.96 (m, 16H), 1.43 (m, 27H); ^13^C NMR (100 MHz, CDCl_3_) δ = 172.9, 172.7, 172.4, 172.3, 167.5, 164.8, 161.1, 152.2, 146.4, 143.5, 137.2, 130.0, 125.8, 124.6, 123.0, 118.0, 111.4, 82.7, 82.4, 82.3, 82.1, 64.3, 58.5, 56.4, 56.3, 56.2, 55.8, 55.6, 54.4, 52.3, 50.7, 50.5, 49.8, 28.0, 27.9 (3C), 27.8 (5C). HR-MS (ESI): *m*/*z* calcd for C_42_H_63_N_7_O_8_F_3_, [M – H]^+^, 850.4690; found 850.4699.

#### 10-Ethyl-[2,2′,2″-(1,4,7,10-tetraazacyclododecane-1,4,7-triyl)triacetic Acid]-(2*E*)-(pyridin-2-yl)-{2-[3-(trifluoromethyl)phenyl]hydrazinylidene}acetate (**L**^**b**^)

Compound (**6**^**b**^) (0.2 g, 0.25 mmol, 1 equiv) was dissolved in 10 mL of (a) DCM and acidified with 5 mL of trifluoroacetic acid (TFA); (b) water and acidified with 5 mL of 36% HCl. Both reactions were stirred for 24 h at rt. Solvents were removed under reduced pressure, and products were additionally freeze-dried to give a yellow powder of 0.17 g (100%).

^1^H NMR (400 MHz, D_2_O) δ = 8.73 (d, *J* = 5.0 Hz, 1H), 8.58 (t, *J* = 8.0 Hz, 1H), 8.42 (d, *J* = 8.0 Hz, 1H), 7.95 (t, *J* = 8.0 Hz, 1H), 7.84 (s, 1H), 7.72 (d, *J* = 8.2 Hz, 1H), 7.57 (t, *J* = 7.9 Hz, 1H), 7.48 (d, *J* = 7.8 Hz, 1H), 3.37 (m, 30H); ^13^C NMR (100 MHz, D_2_O) δ = 161.3, 147.3, 146.1, 142.1, 140.8, 131.4, 131.0, 130.3, 126.5, 125.2, 124.9, 122.5, 121.6, 119.8, 117.8, 115.0, 112.8, 66.0, 56.4, 55.8, 54.7, 53.7, 51.2, 51.0, 50.8, 50.6, 50.3, 48.6, 48.3. HR-MS (ESI): *m*/*z* calcd for C_30_H_39_N_7_O_8_F_3_, [M – H]^+^, 682.2813; found 682.2801.

#### **L**^**b**^ Complexes

Water solution of **L**^**b**^ (1 equiv) and LnCl_3_/LnNO_3_ hexahydrate (1.1 equiv) was stirred at room temperature for 24 h. Then, pH was maintained between 5.0 and 6.0 by 0.1 M of aqueous NaOH and stirred for 7 days. The pH of the mixture was increased to 10–11 to precipitate excess Ln^3+^ as Ln(OH)_3_. After removing the precipitate by filtration, the remaining filtrate was freeze-dried. MS data of all obtained compounds are shown in Table 1.

**Table 1:** HR-MS (ESI) Results and Appearance of the Resulting Compounds

	*m*/*z*		
compound formula	calculated	found	appearance
C_30_H_36_N_7_O_8_F_3_Eu	832.1793	832.1799	Yellow powder
C_30_H_36_N_7_O_8_F_3_Ho	844.1880	844.1898	Yellow powder
C_30_H_36_N_7_O_8_F_3_Dy	843.1877	843.1852	Yellow powder
C_30_H_36_N_7_O_8_F_3_Gd	837.1826	837.1823	Yellow fluffy powder
C_30_H_36_N_7_O_8_F_3_Er	847.1909	847.1928	Yellow powder
C_30_H_36_N_7_O_8_F_3_Nd	823.1685	823.1691	Yellow solid
C_30_H_36_N_7_O_8_F_3_La	818.1641	818.1672	Yellow powder
C_30_H_36_N_7_O_8_F_3_Cr	731.1983	731.1966	Green powder
C_30_H_36_N_7_O_8_F_3_Yb	853.1971	853.1972	Yellow solid
C_30_H_36_N_7_O_8_F_3_Pr	820.1654	820.1660	Yellow solid
C_30_H_36_N_7_O_8_F_3_Ce	819.1632	819.1632	Yellow fluffy powder
C_30_H_37_N_7_O_8_F_3_Cu	743.1952	743.1957	Green powder
C_30_H_36_N_7_O_8_F_3_Fe	735.1927	735.2029	Orange oily solid
C_30_H_36_N_7_O_8_F_3_Co	739.1988	739.2011	Orange powder

### Compound Characterization

The products were characterized by ^1^H and ^13^C NMR in CDCl_3_ or D_2_O, while ^19^F NMR spectra were recorded in aqueous solution (10% D_2_O). The spectra were referenced internally using residual protonated solvent resonances relative to tetramethylsilane (δ = 0 ppm) or trifluoroacetic acid (^19^F NMR, δ = −76.5 ppm) as an internal standard. *T*_1_ and *T*_2_ measurements were performed using inversion recovery and the CPMG sequences (16 × 3 data points), respectively. Samples of complexes for ^19^F NMR relaxation experiments were prepared by mixing 500 μL of aqueous solution (30 mmol dm^–3^) with 50 μL of D_2_O. The pH was controlled by the addition of 0.1 mM TFA or HCl and 0.1 mM NaOH. An Agilent 400-MR instrument was used for all NMR experiments. To conduct the ^19^F MRI experiments, we acidified the samples with 0.1 mM HCl and avoided the use of TFA during synthesis to prevent interference from the ^19^F NMR signal in the measurements. HR-MS studies were performed with a Xevo G2 QTof instrument (Waters) equipped with an ESI source. A pHenomenal MD 8000L pH meter with a standard glass electrode was used for the pH measurements (3.0 M KCl solution was used as a reference to pH = 7).

### MRI Experiments

MRI data were acquired on a horizontal 9.4 T BioSpec 94/20 preclinical scanner (Bruker, Germany) running PV5.1 software, equipped with a B-GA12SHP gradient coil and a T20013V3 (Bruker) double-tuned (^1^H and ^19^F) transmit–receive radiofrequency coil. For MR imaging, four solutions were prepared in standard NMR vials (300 μL) with a pH of 7.0, 5.0, 4.5, and 4.0. The pH of the solutions was changed by adding the appropriate amount of HCl (1–10 μL). Initial scanner adjustments were completed using ^1^H frequency; then, the RF was tuned to the ^19^F frequency of the CF_3_ group. The correct setting of the ^19^F resonance frequency was verified by acquiring the NMR spectrum with the single pulse method. Subsequent images were obtained using this frequency, thus using the fluorine signal from CF_3_ groups. To show the influence of pH on relaxation times, several images were taken using the gradient echo fast low angle shot (FLASH) method, changing the sequence parameters: echo time (TE) and repetition time (TR). Additionally, a *T*_2_-weighted image was acquired with the rapid acquisition with relaxation enhancement (RARE) method as well. Geometric parameters of image acquisition were as follows: signal intensity (SI) 9 mm, field of view (FOV) 25 mm^2^, matrix acquisition (MTX) 32. FLASH images were acquired with the following timing parameters TE: 1.8, 1.8, 6 ms; TR: 6, 100, 100 ms, respectively, and FA 50°. RARE image was acquired with TE 13 ms and TR 100 ms.

### Computational Methods

All density-functional theory (DFT) calculations were performed using the Orca 4.2.1. Full geometry optimizations of L complexes were performed in aqueous solution employing the hybrid metageneralized gradient approximation, with the TPSSh exchange–correlation functional.^50^ In these calculations, an energy-consistent large core quasi-relativistic ECP (LCRECP) and its associated [5s4p3d]-GTO valence basis set for lanthanoid were employed, while the ligand atoms and other metals were described using the standard 6-31G(d) basis set. The input files and molecular plots were prepared with Avogadro software.^51^

## Results and Discussion

### Design and Synthesis

**Figure 1:**
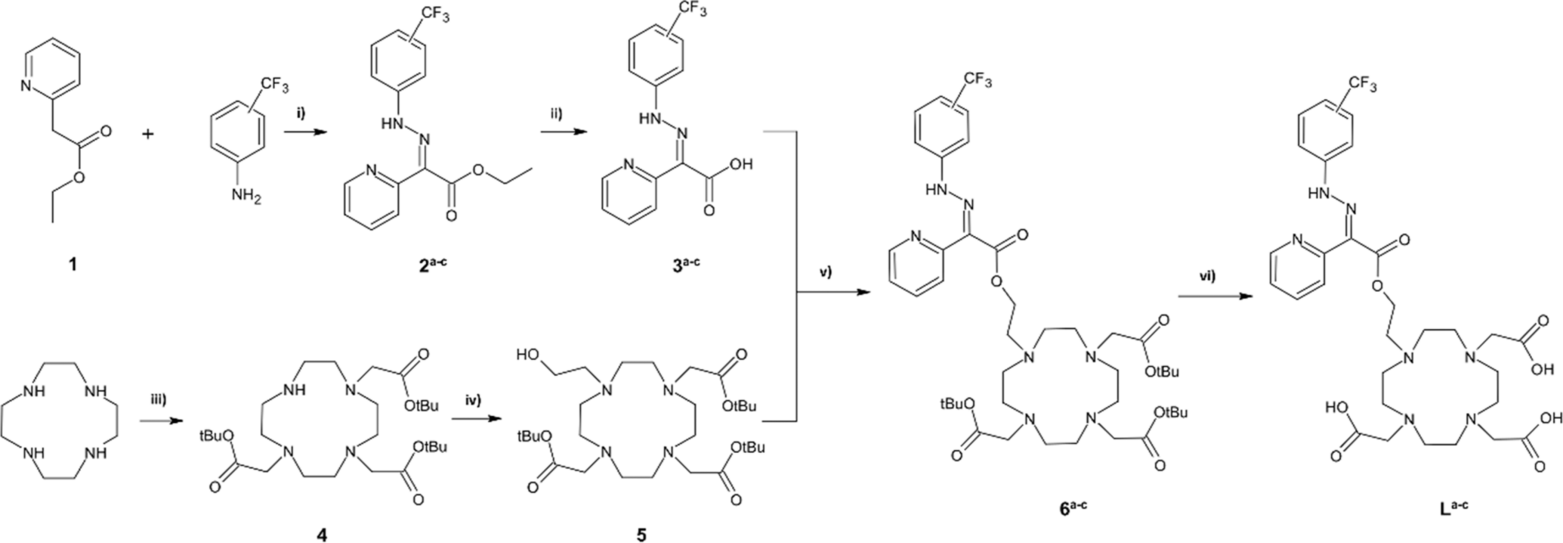
General synthetic path for **L**^**a-c**^ ligand series: (i) HCl, EtOH, NaOAc, 0 °C, 8 h; (ii) KOH, MeOH, 35 °C, 2 h; (iii) BrCH_2_COOtBu, Na_2_CO_3_, ACN, 0 °C, 24 h; (iv) BrCH_2_CH_2_OH, K_2_CO_3_, ACN, rt, 7 days; (v) DCC, DMAP, DCM, rt, 14 days; and (vi) TFA/HCl, DCM/water, rt, 24 h.

Our previous work revealed that hydrazone-based switches with a CF_3_ reporting group are suitable for pH imaging.^9^ The signal readout changes relied on the chemical shift changes in ^19^F and ^1^H NMR spectra induced by *E*/*Z* isomerization of the hydrazone moiety. We also noticed that the relaxation times of fluorine signals do not change under the isomerization. It was interesting to investigate whether the hydrazone switch architecture could be adapted for pH imaging using relaxation rate changes instead of chemical shift. This would allow for the use of standard *T*_1_- or *T*_2_-weighted image acquisitions and ensure consistency, diagnostic accuracy, efficiency, and effective communication in medical imaging. A possible solution (Figure 1) was to introduce a paramagnetic center into the switch, either through substitution of one of the aromatic rings or the ethyl functional group with a paramagnetic complex. The latter was the most straightforward solution due to the facile hydrolysis and esterification of the obtained acid with a DOTA-derived 2-bromo ethyl alcohol **5**. Deprotection of the ester **6**^**b**^ with trifluoroacetic acid or HCl followed, leading to the desired product. The obtained ligand **L**^**b**^ remained stable at pH levels between 2 and 3 for at least 1 week.

**Figure 2:**
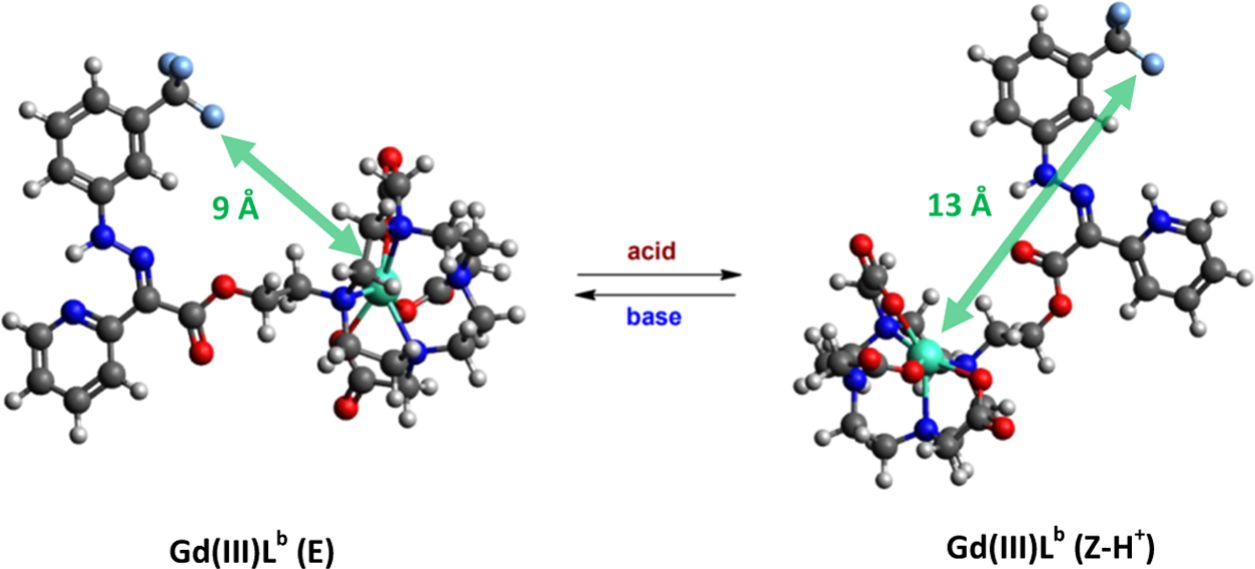
DFT-optimized structures during isomerization of Gd(III)L^b^ complex and the ^19^F–Gd(III) distances.

There were three possible variants of the ligand L, with the CF_3_ group in either the ortho (L^a^), meta (L^b^), or para (L^c^) position. Using the geometric TPSSh/ECP(LCRECP)/6-31G(d) optimization of the corresponding Gd(III) complexes of L^a-c^, the changes in the fluorine-paramagnetic ion distances (^19^F–M) during *E*/*Z* isomerization of each putative switch (Figure 2) were estimated. Comparing the meta isomer to the other variants, it was found that the ^19^F–M distance changes were much smaller (Table 2). Furthermore, among the isomeric hydrazone switches 2^a-c^, it was observed that the chemical shift of the ^19^F signal in the meta isomer only changed by 0.1 ppm upon acidification, in contrast to 1.8 and 0.8 ppm for the ortho and para isomers, respectively.^9^ No changes in chemical shifts of the CF_3_ reporter were expected in L^b^ due to the nearly identical 60° angle between the fluorines and the plane of the complex for either E and Z–H^+^ form and relatively large (9–13 Å) ^19^F–M distance. Therefore, we focused solely on the meta isomer (L^b^), as it offered the highest potential changes in relaxation rates due to the PRE effect and a stable position of the ^19^F signal, allowing for the tracking of a single narrow ^19^F resonance for imaging purposes.

**Table 2:** Calculated ^19^F–M Distances in Isomeric Gd(III) Complexes of L^a-c^^a^

	^19^F–M distance		
form	L^a^ (Å)	L^b^ (Å)	L^c^ (Å)
E	6.88	9.01	12.20
Z–H^+^	7.61	12.93	13.08
absolute change	–0.73	–3.92	–0.88

a The distances were calculated from DFT-optimized structures.

The selection of the most suitable paramagnetic ion for complexation was conducted by theoretical calculations based on the Bloch–Redfield–Wangsness (BRW) theory, taking into account the electron–nucleus dipole–dipole and Curie interactions only. This was justified by an earlier review of available ^19^F relaxation data for DOTA-derived paramagnetic complexes.^52^ The highest relaxation time differences from pH-induced isomerization resulted from complexes of Gd(III), Ho(III), Dy(III), Er(III), Cu(II), or Cr(III) (Figure S18 and Table S1).

### ^19^F NMR Experiments

**Figure 3:**
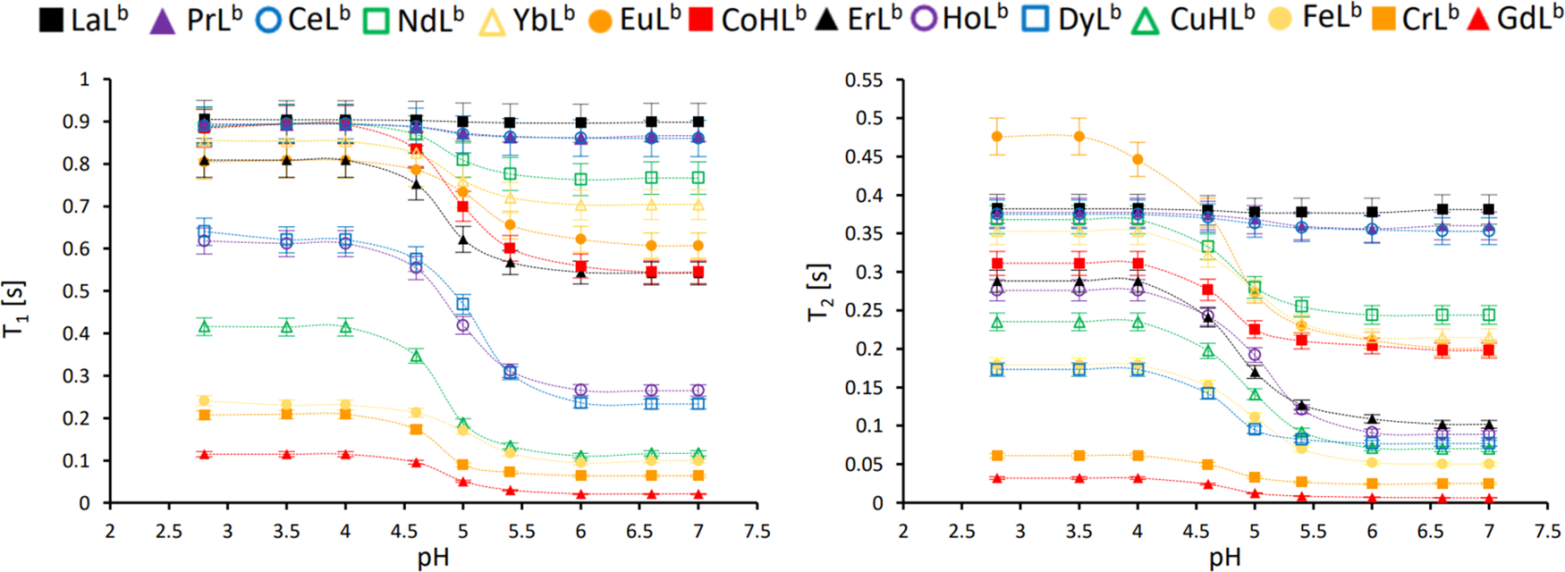
Relationship between the pH and observed *T*_1_ and *T*_2_ relaxation times of the CF_3_ group in various complexes of L^b^. The error bars represent the standard deviations.

The results of the theoretical predictions were verified experimentally for selected metal ions. The complexes were well-soluble (at least 50 mg mL^–1^) in water, allowing for detailed studies of pH-induced relaxation time changes in buffered aqueous solutions. In each case, the solution of a complex was acidified by varying amounts of 0.01–1.00 mM TFA to achieve nine values of pH (7.0, 6.6, 6.0, 5.4, 5.0, 4.4, 4.0, 3.5, 2.8), as shown in Figure 3.

**Figure 4:**
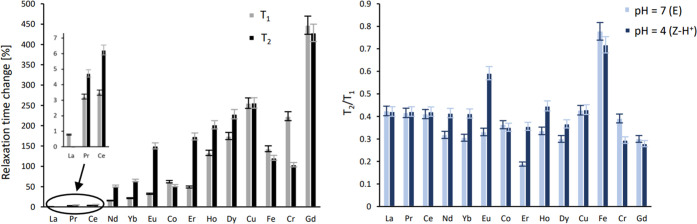
Relative change of the relaxation time of ^19^F in *E* and *Z* isomers of various L^b^ complexes and corresponding *T*_2_/*T*_1_ ratios.

Except for the reference diamagnetic lanthanum complex (Figure 3, black squares) and the free ligand L^b^ (*T*_1_ = 908 ± 27 ms and *T*_2_ = 370 ± 14 ms), a gradual increase in *T*_1_ and *T*_2_ relaxation times with decreasing pH was observed. This indicates that the CF_3_ group was moving away from the paramagnetic center (pendant DOTA-complex) during the transformation from the E to the Z–H^+^ isomer. For all investigated complexes, movement from the CF_3_ group away from the paramagnetic center occurred in the pH range of 5.5–4.0. In terms of the absolute change for *T*_1_, the highest results were observed for Dy(III)L^b^ and Ho(III)L^b^ (Δ*T*_1_ = 407 ± 27 and 353 ± 20 ms, respectively) and the lowest for Ce(III)L^b^ and Pr(III)L^b^ (Δ*T*_1_ = 30 ± 2 and 29 ± 2 ms). With regard to *T*_2_, the highest changes were observed for Eu(II)L^b^ and Er(III)L^b^ (Δ*T*_2_ = 270 ± 11 and 186 ± 8 ms, respectively) and the lowest for Ce(III)L^b^ and Pr(III)L^b^ (Δ*T*_2_ = 22 ± 1 and 17 ± 2 ms). Regarding the relative changes in relaxation times, which are more significant for imaging purposes, Gd(III)L^b^ displayed the best properties with a 450 ± 26% increase in *T*_1_ and a 430 ± 25% increase in *T*_2_ during isomerization between E and Z–H^+^ isomers (Figure 4).

Gd(III)L^b^ displayed the smallest overall relaxation times (110 ms for *T*_1_ and 50 ms for *T*_2_), allowing for the most rapid signal acquisition. A small disadvantage of the Gd(III)L^b^ complex is the low *T*_2_/*T*_1_ ratio, which results in line broadening. The ratio was similar (0.3–0.4) for most other complexes, except for Fe(III)L^b^ where it reached 0.8.

The reversibility of the transition was investigated by repeatedly acidifying the HoL^b^ complex solution (500 mL, 30 mM) sample to pH = 3 with 1 mM TFA and neutralizing with 1 mM NaOH. This allowed for minimal changes in the concentration of the sample. The process was repeated 5 times (Figure S15). The complex showed stability after all cycles. Although the complex precipitated at pH > 12, this was not considered relevant because typical operating conditions are at pH 4–7.

### ^1^H NMR Experiments

Due to the presence of the DOTA-derived paramagnetic center, the switch has the potential to be used as a ^1^H MRI imaging probe. To test this, only the Gd(III)L^b^ complex was studied at concentrations ranging from 0.0 to 2.0 mM as a *T*_1_- and *T*_2_-relaxation agent. A water signal at 4.7 ppm was observed at pH values 7 and 4. Regardless of the pH, the impact of the concentration of the complex was identical. This suggested that the water exchange in the coordination sphere is not affected by the pH, presumably due to the significant distance of the metal from both aromatic rings of the hydrazone moiety that is not affected during isomerization (Figure S25). At a concentration of 2.0 mM of Gd(III)L^b^, the values *R*_1_ = 8.5 Hz and *R*_2_ = 2.6 Hz were obtained corresponding to the relaxivity values of *r*_1_ = 2.91 mol^–1^ s^–1^ and *r*_2_ = 1.01 mol^–1^ s^–1^ (Figure S16). These values are similar to those observed for GdDOTA, Gd-DTPA, and Gd-DO3A-butrol.^53^ Therefore, the Gd(III)L^b^ complex can be used as a bimodal contrast agent with the properties of a standard ^1^H contrast agent under ^1^H MRI and the ability to act as a pH-sensitive agent under ^19^F MRI.

### MRI Experiments

The final confirmation of the suitability of the Gd(III)L^b^ complex as a ^19^F MRI agent was performed using a 9.4 T MRI scanner. Four samples containing a 10 mM solution (300 mL) of Gd(III)L^b^ at pH 7.0, 5.0, 4.5, and 4.0 were used as phantoms. The number of repetitions was set so that the acquisition time for each image was approximately 60 min, with 19,000, 1125, 1125, and 4500 repetitions for images 1, 2, 3 (FLASH), and 4 (RARE), respectively. The images obtained showed a strong correlation between pH and MRI contrast, particularly in the *T*_2_ RARE sequence due to the dominant role of the transverse relaxation time *T*_2_ in image contrast. The reader interested in a more detailed analysis of the contrast in the RARE sequence is referred to the original paper by Hennig and coauthors.^54^

**Figure 5:**
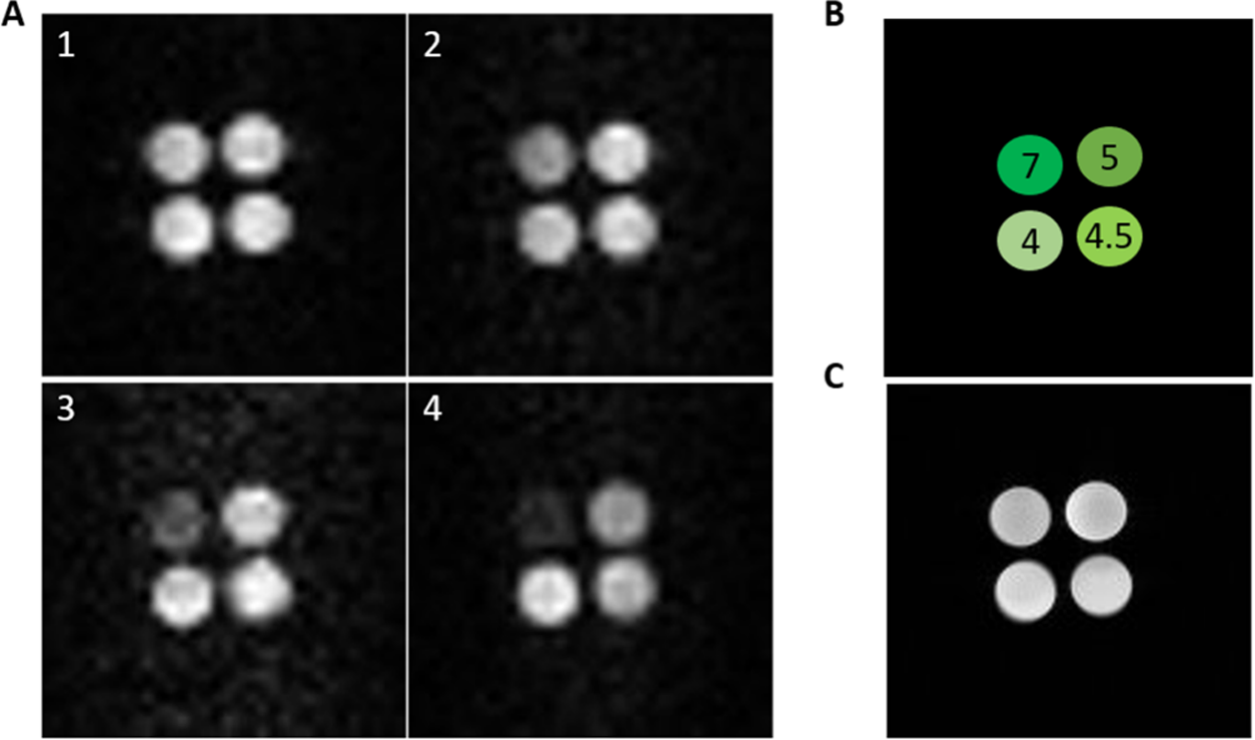
(A) ^19^F magnetic resonance imaging (MRI) of pH gradient (pH 7.0, 5.0, 4.5, 4.0) of 10 mM Gd(III)L^b^ under different imaging conditions: 1, 2 (*T*_1_) and 3 (*T*_2_) fast low angle shot images (FLASH); 4 (*T*_2_) rapid acquisition with relaxation enhancement image (RARE). (B) arrangement of samples in the apparatus. (C) ^1^H MRI. Field of view: 25 mm^2^, matrix acquisition: 32.

In Figure 5A4, the pH 7 phantom (*T*_2_ = 6 ms) appears darker than the pH 4 phantom (*T*_2_ = 32 ms), indicating a directly proportional relationship between pH and MRI image brightness. The pH–MRI contrast relationship was not as obvious as for the RARE images since FLASH images are generally sensitive to both *T*_1_ and *T*_2_/*T*_1_ ratios. The data presented in Figure 3 demonstrates that alterations in pH have a comparable impact on *T*_1_ and *T*_2_ values of these complexes. Consequently, the *T*_2_/*T*_1_ ratio remains stable irrespective of pH, resulting in images that primarily emphasize *T*_2_ contrast.

### Properties of ML^b^ from the Point of View of the Bloch–Redfield–Wangsness Relaxation Theory

The initial calculations of relaxation properties for ML^b^ complexes for design purposes were performed assuming a constant rotational correlation time of 0.25 ns and distances of 9 and 13 Å for the E and Z–H^+^ isomers, respectively, as derived from DFT calculations of Gd(III) complexes. The relative predicted suitability of selected ML^b^ complexes was found to be consistent with observed values, as shown in Figures S18 and S19. However, the measured relaxation times differed from the predicted values in most cases by approximately 10% or more.

In the next step, the rotational correlation time and the ^19^F–M distance were optimized to seek the best agreement between the observed and predicted *T*_1_ and *T*_2_ relaxation times. The result was a ^19^F–M distance in E isomers of 8.7 Å (for lanthanoids) or 8.3 Å (for divalent transition metals) and 13.4 Å in Z–H^+^ isomers, with a rotational correlation time of 0.33 ns. The deviations between predicted and observed data were not higher than 5%, indicating that the assumption that only dipole–dipole and Curie mechanisms contribute to the observed relaxation rates was justified (Figures S19 and S20).

The accuracy of the BRW theoretical predictions was further improved by allowing the distance for each type of paramagnetic ion to vary, as demonstrated in Figure S21. The distances predicted from relaxation data were compared with DFT distances calculated for each complex in both E and Z–H^+^ configurations. In all cases, except for the Eu(III)L^b^ complex, >99% agreement was achieved, indicating the suitability of the BRW model for predicting the properties of ^19^F contrast agents (Figures S22–S24 and Table S2). The discrepancy between the predicted and observed values for the Eu(III)L^b^ complex is likely due to the significant chemical shift anisotropy–anisotropic dipolar shielding cross-correlation (CSA × DSA) for this ion.^52,55^ When this effect is taken into account in the BRW model, the calculated angle between the principal axes of the chemical shift anisotropy and dipolar shielding anisotropy tensors is 90° for the Eu(III)L^b^ complex. For the other complexes, in which the cross-correlation effect is negligible, the angle is 64°.

## Conclusions

In summary, this study aimed to investigate a series of hydrazone-based fluorinated molecular switches with pendant paramagnetic groups as potential ^19^F MRI and ^1^H contrast agents. By positioning the paramagnetic group at 9–13 Å from the CF_3_ group, we observed changes in *T*_1_ and *T*_2_ relaxation times due to the varying PRE effect of the metal ion as a result of *E*/*Z* isomerization induced by pH changes. The most suitable complex for MR imaging was found to be Gd(III)L^b^, leading to over 400% relaxation time changes in the pH range of 4–7. We also investigated other metal ions in terms of theoretical calculations, which were verified by experimental results showing the reliability of BRW theoretical predictions. The isomerization process can be tracked solely on the basis of relaxation time differences using *T*_2_-weighted imaging sequences due to the negligible chemical shift changes of the CF_3_ group in the ^19^F NMR spectrum. This is due to the lack of pseudocontact shift effects and the minimal impact of isomerization on the chemical shift of the CF_3_ group in the meta position. The use of such probes in ^19^F MRI can be adapted for the detection of specific molecular and physiological events, such as changes in pH,^56^ the presence of tumor tissues,^57^ or enzymes,^58^ which are not visible with ^1^H MRI of these probes. Additionally, Gd(III) complexes with similar structures are widely used in medical diagnostics and are considered safe at small doses.^59^ The aromatic hydrazones with a similar structure were found to be weakly mutagenic but not carcinogenic, exhibiting good renal excretion and low toxicity toward healthy tissues and cells.^60^ This suggests the potential usefulness of the proposed probes *in vivo*, which we will study further.

## Supporting Information Available

Additional experimental details and methods of NMR characterization, including the synthesis, ^1^H, ^13^C, and ^19^F NMR spectra, relaxation studies, and DFT results (PDF)
