# Supplementary material for: Hydrazone Molecular Switches with Paramagnetic Center as 19F Magnetic Resonance Imaging Relaxation Enhancement Agents for pH Imaging
Source: ACS Sens. 2023 May 18;8(5):1971–9. doi: 10.1021/acssensors.3c00080 (PMC10226166; doi:10.1021/acssensors.3c00080)
Supplement: Supplementary file 1 — se3c00080_si_001.pdf [file se3c00080_si_001.pdf]

Supporting information

Hydrazone molecular switches with paramagnetic center as ^{19}F magnetic resonance imaging relaxation enhancement agents for pH imaging

Dawid Janasik[a], Krzysztof Jasiński[b], Julia Szreder[a], Władysław P. Węglarz[b], Tomasz Krawczyk[a*]

tomasz.krawczyk@polsl.pl

[a] Department of Chemical Organic Technology and Petrochemistry, Silesian University of Technology, 44-100 Gliwice, Poland

[b] Institute of Nuclear Physics Polish Academy of Sciences, 31-342 Krakow, Poland

NMR spectra

(**1**) Ethyl 2-(pyridin-2-yl)acetate

(**2**[b]) Ethyl (2E)-(pyridin-2-yl) {2-[3-(trifluoromethyl)phenyl]hydrazinylidene}acetate

(**3**[b]) (2E)-(pyridin-2-yl){2-[3-(trifluoromethyl)phenyl]hydrazinylidene}acetic acid

(**4**) Tert-butyl 2,2',2"-(1,4,7,10-tetraazacyclododecane-1,4,7- triyl)triacetate

(**5**) Tert-Butyl 2,2',2"-(10-(2-hydroxyethyl)-1,4,7,10-tetraazacyclododecane-1,4,7-triyl)triacetate

(**6**[b]) 10-ethyl-[tert-Butyl 2,2',2"-(1,4,7,10-tetraazacyclododecane-1,4,7-triyl)triacetate]-(2E)-(pyridin-2-yl) {2-[3-(trifluoromethyl)phenyl]hydrazinylidene}acetate

(**L**[b]) 10-ethyl-[2,2',2"-(1,4,7,10-tetraazacyclododecane-1,4,7-triyl)triacetic acid]-(2E)-(pyridin-2-yl) {2-[3-(trifluoromethyl)phenyl]hydrazinylidene}acetate

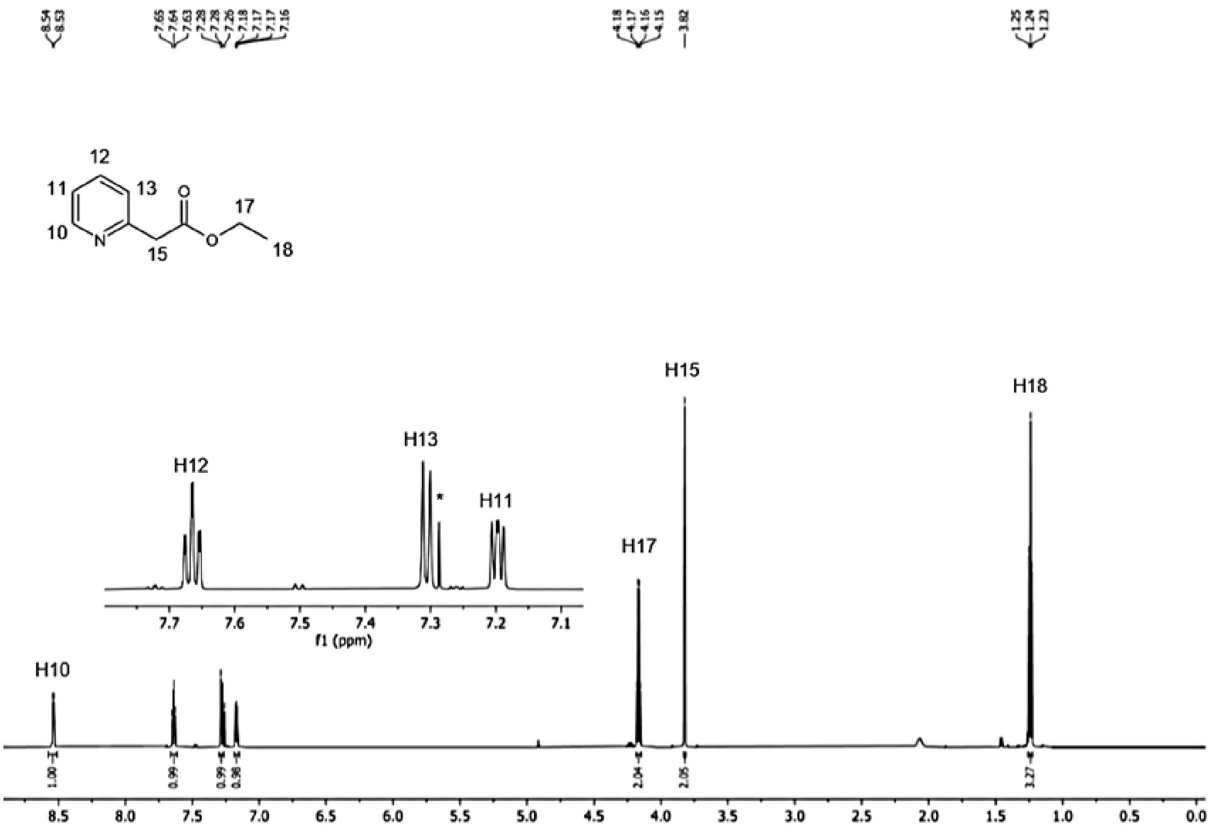

Figure S1. ^{1}H NMR spectrum of (**1**) in $CDCl_3$ at 298 K.

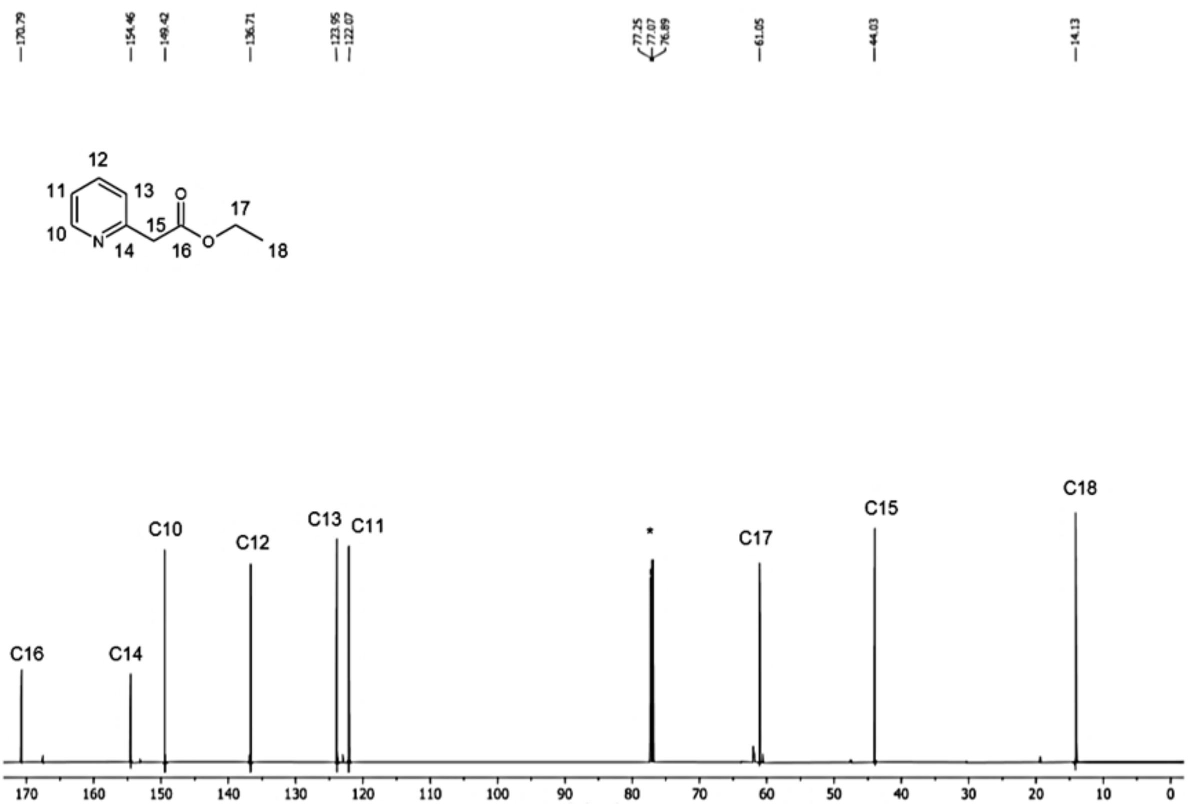

Figure S2. ^{13}C NMR spectrum of (**1**) in $CDCl_3$ at 298 K.

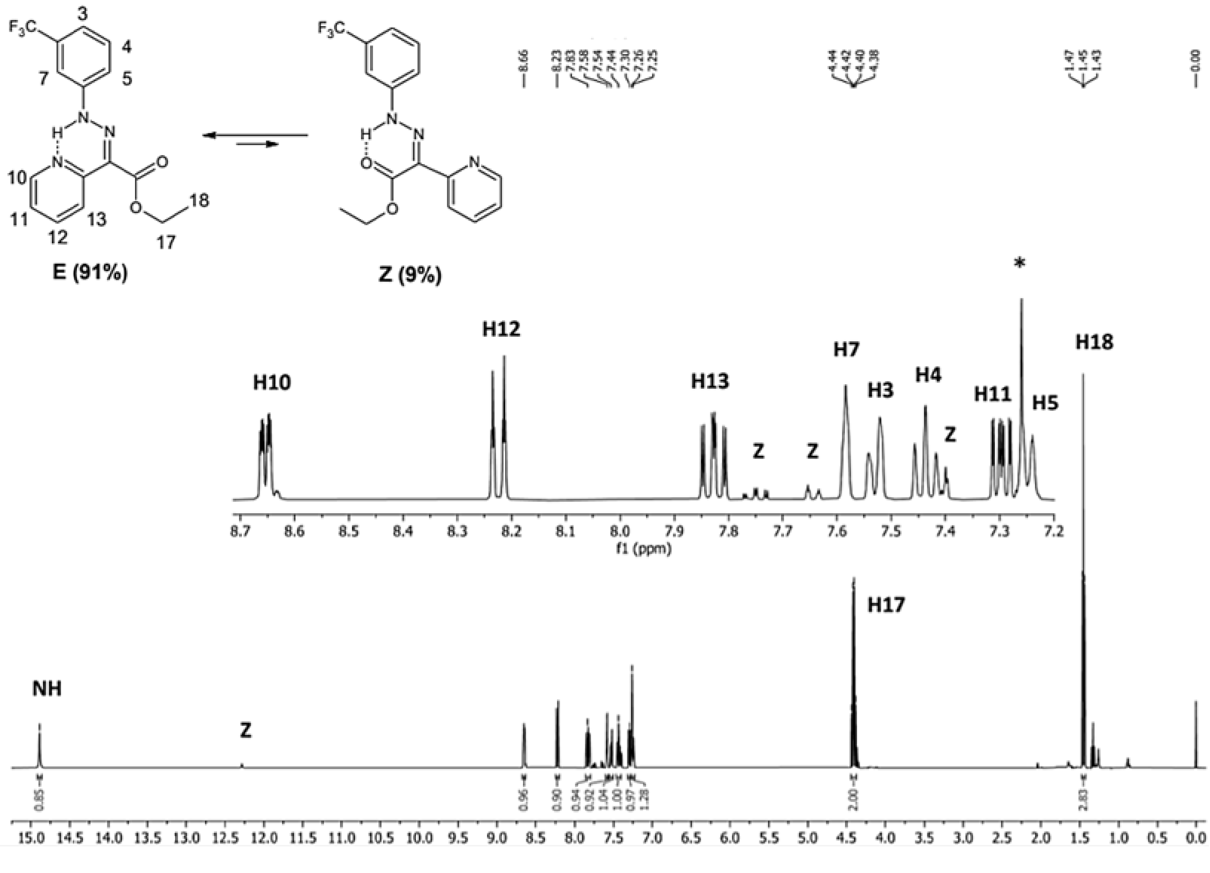

Figure S3. ^{1}H NMR spectrum of (2^b) in $CDCl_3$ at 298 K.

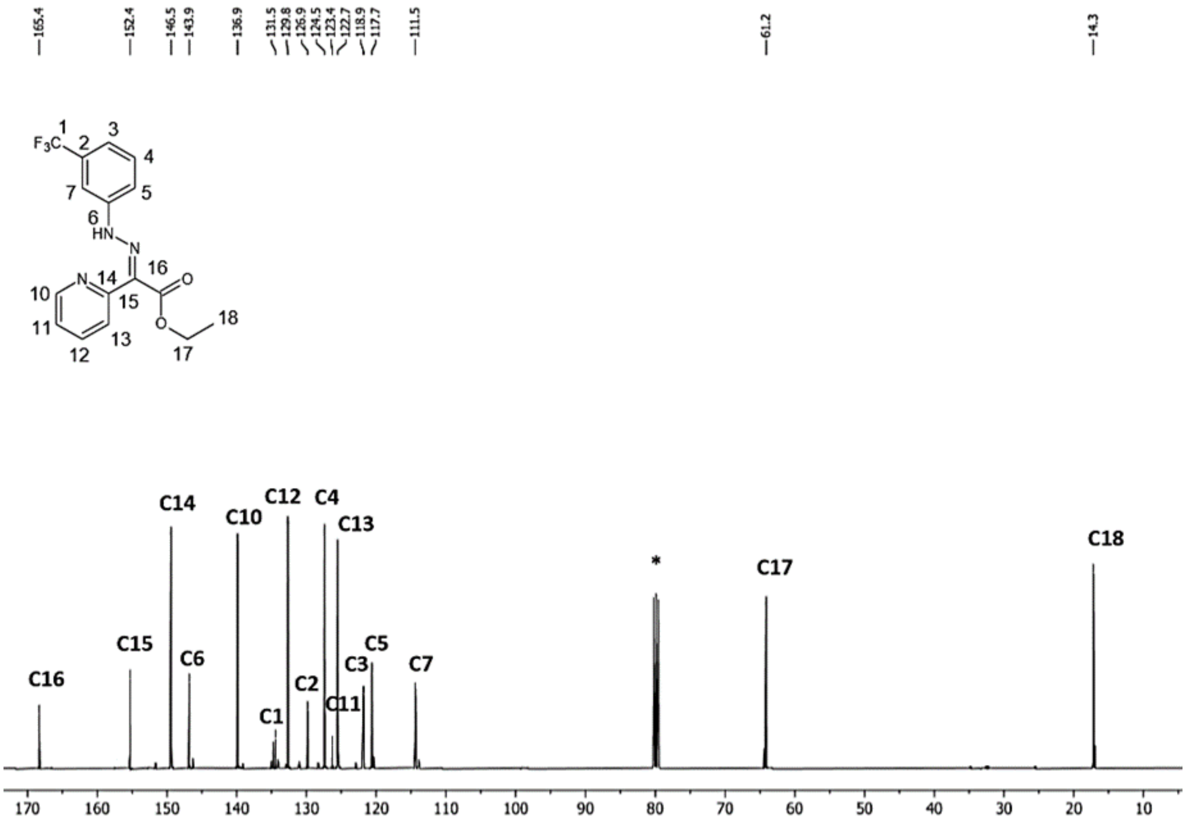

Figure S4. ^{13}C NMR spectrum of (**2**[b]) in $CDCl_3$ at 298 K.

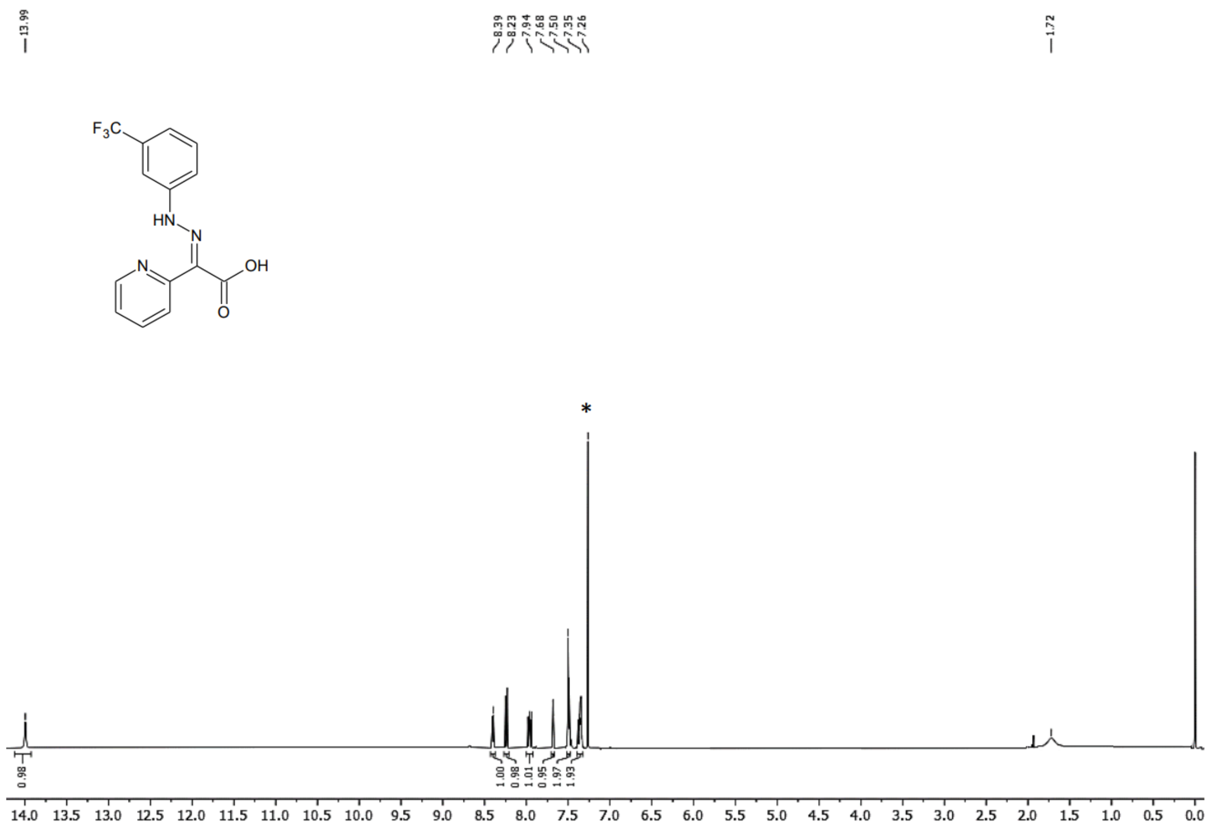

Figure S5. ^{1}H NMR spectrum of (**3**[b]) in $CDCl_3$ at 298 K.

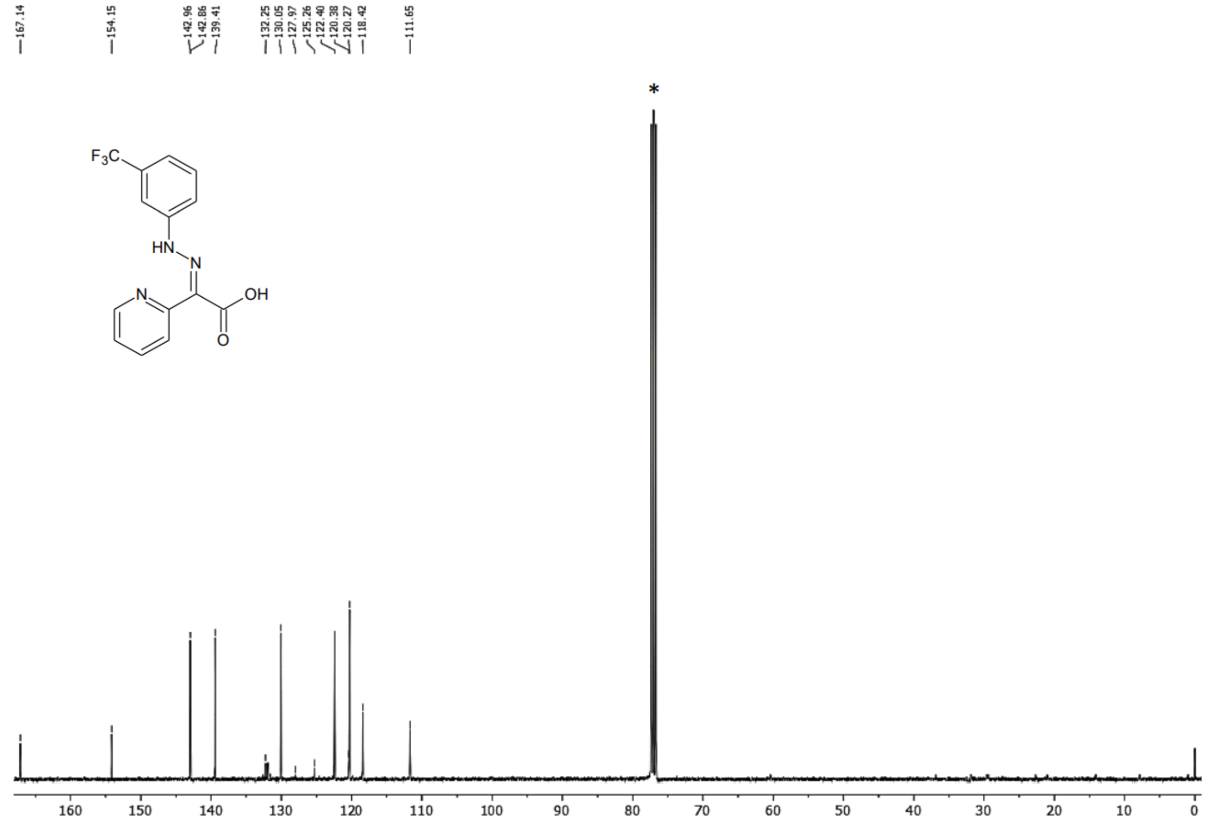

Figure S6. ^{13}C NMR spectrum of (**3**[b]) in $CDCl_3$ at 298 K.

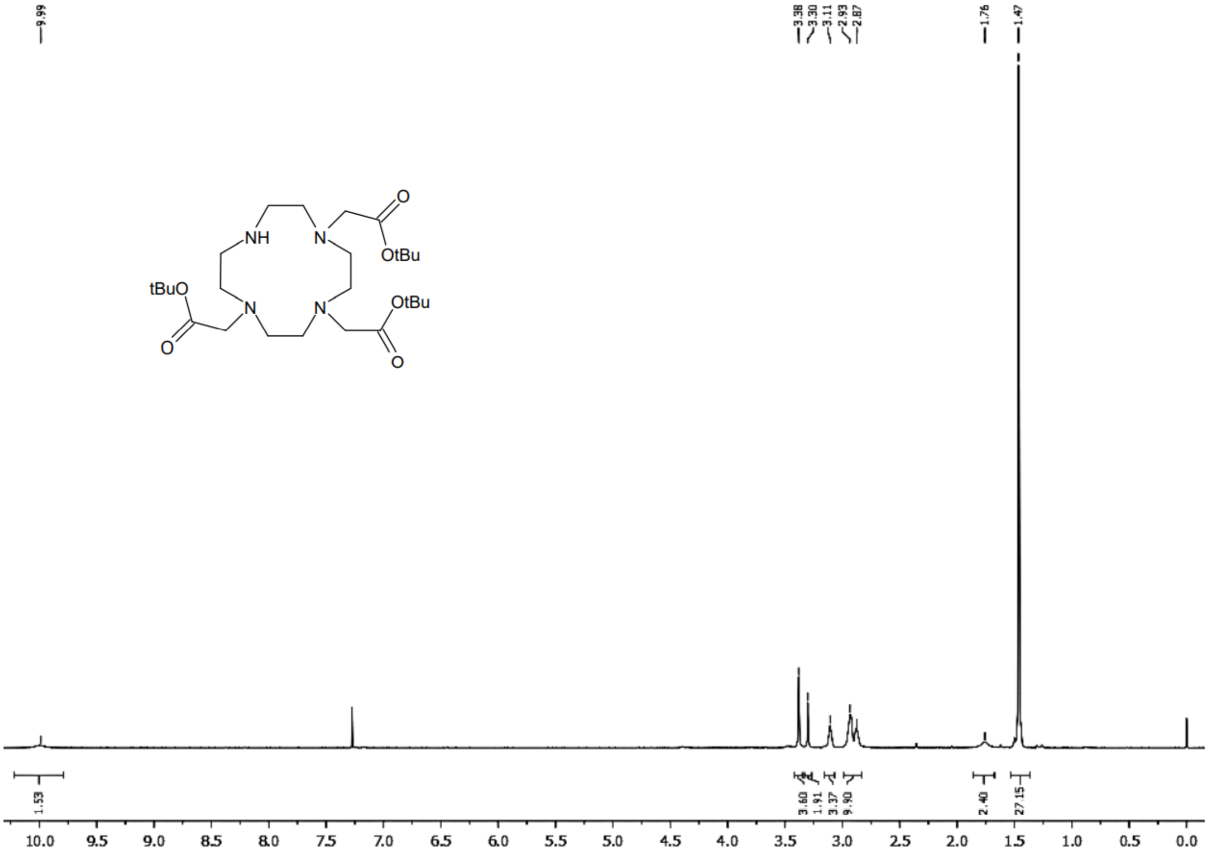

Figure S7. ^{1}H NMR spectrum of (**4**) in $CDCl_3$ at 298 K.

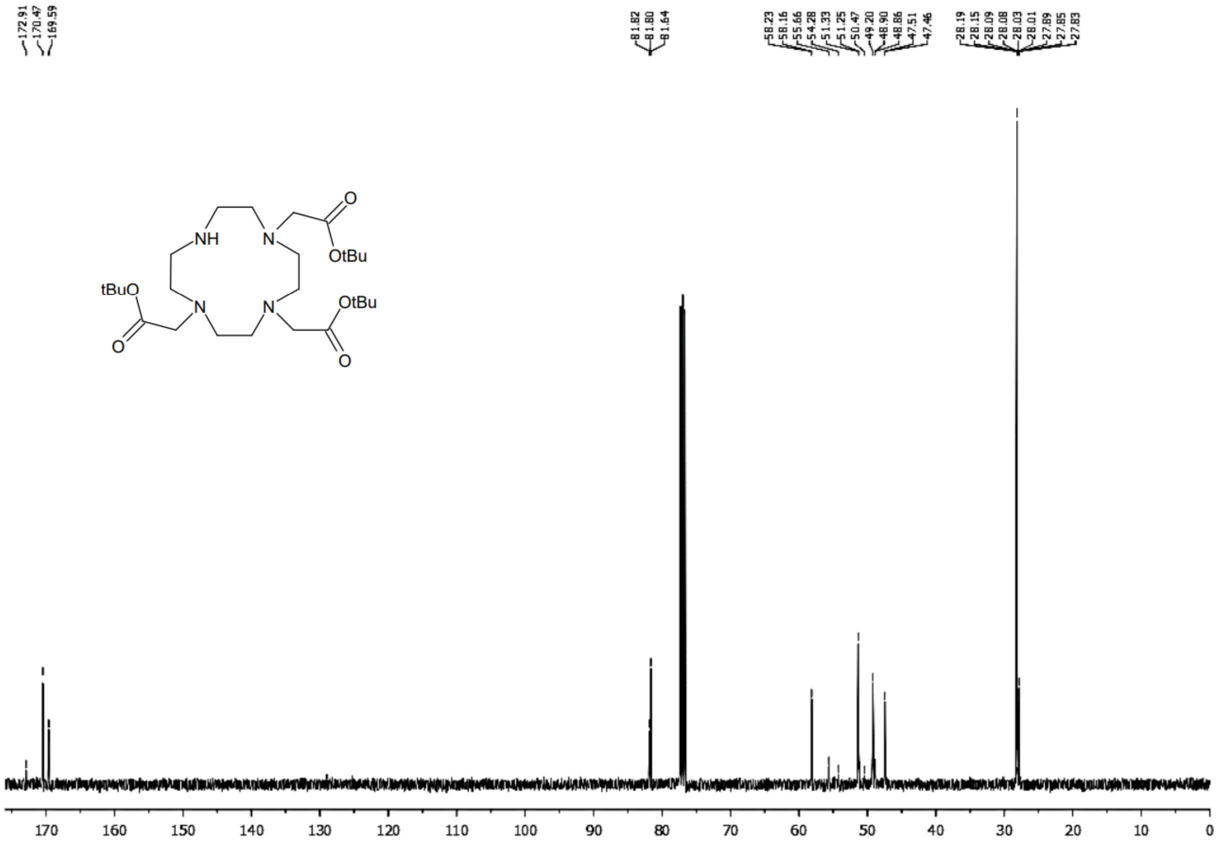

Figure S8. ^{13}C NMR spectrum of (**4**) in $CDCl_3$ at 298 K.

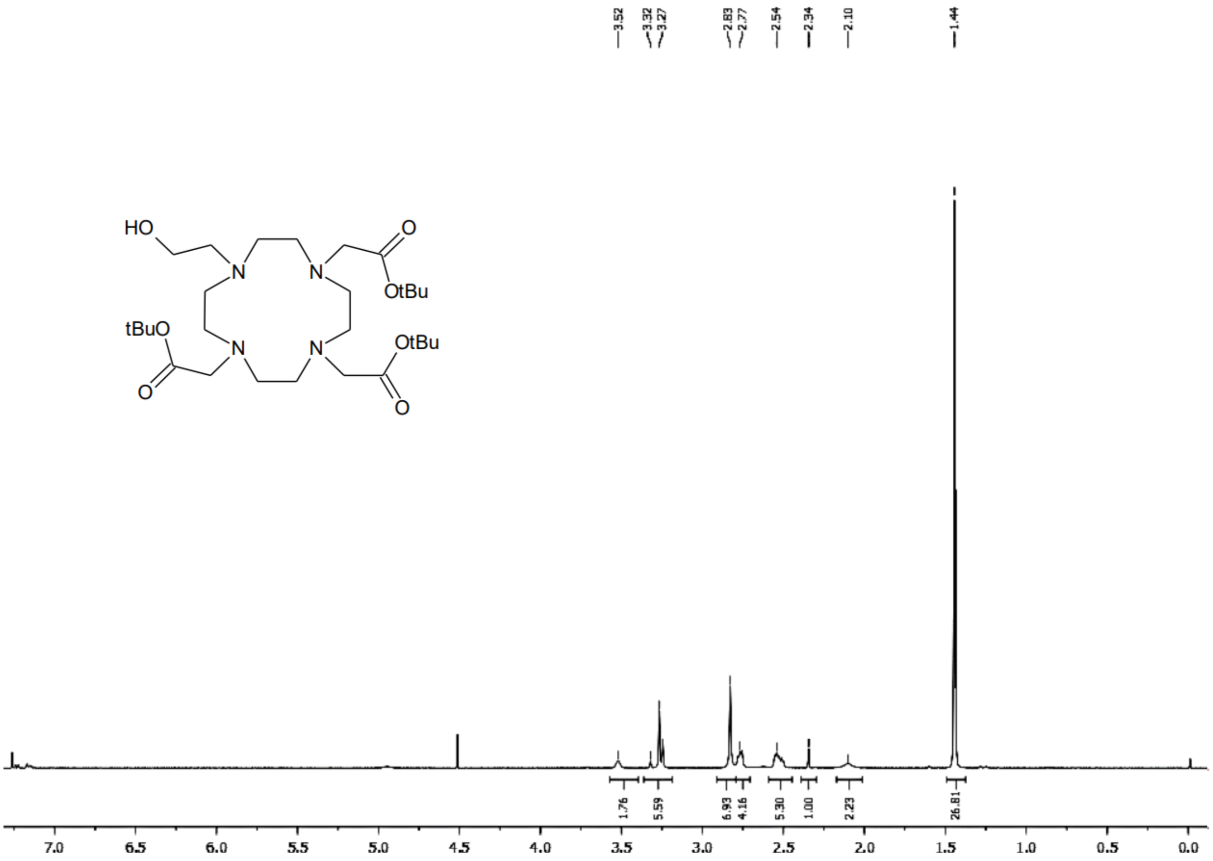

Figure S9. ^{1}H NMR spectrum of (**5**) in $CDCl_3$ at 298 K.

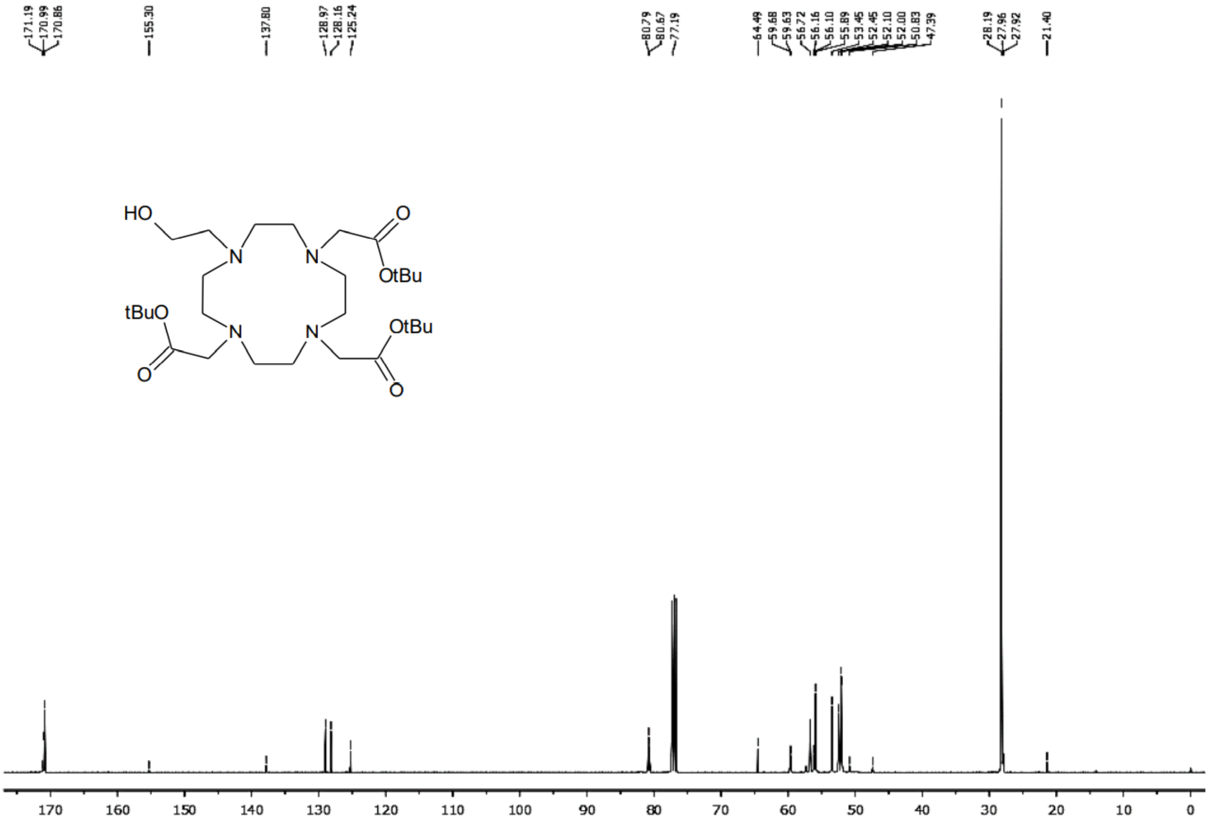

Figure S10. ^{13}C NMR spectrum of (**5**) in $CDCl_3$ at 298 K.

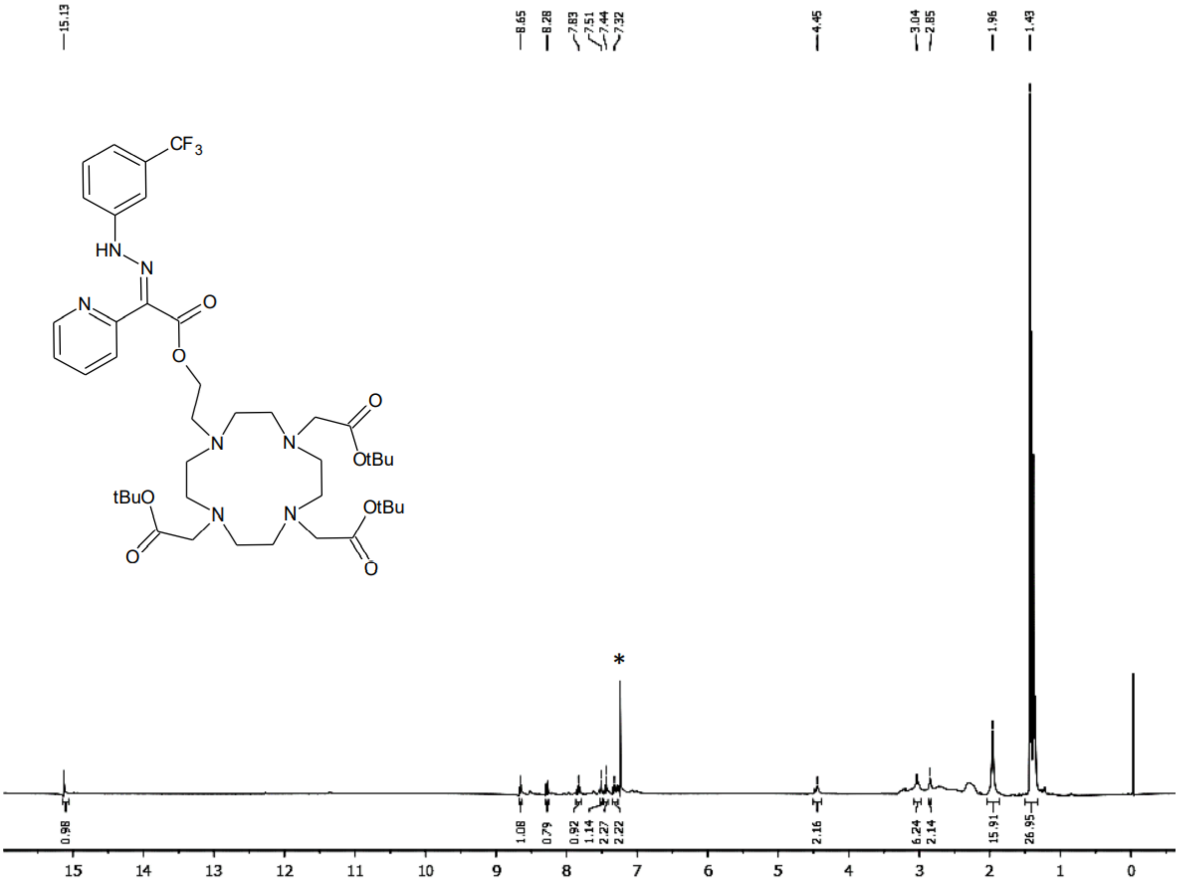

Figure S11. ¹H NMR spectrum of (**6**[b]) in $CDCl_3$ at 298 K.

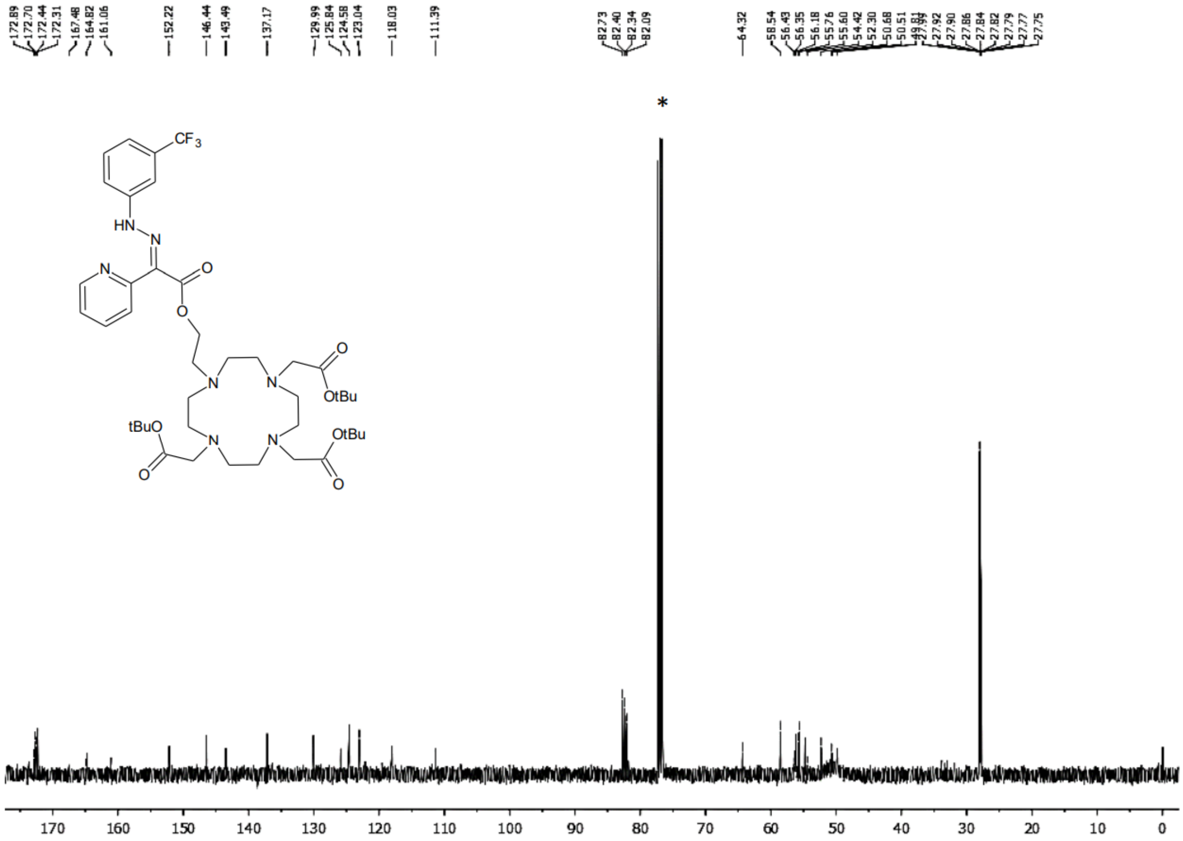

Figure S12. ¹³C NMR spectrum of (**6**[b]) in $CDCl_3$ at 298 K.

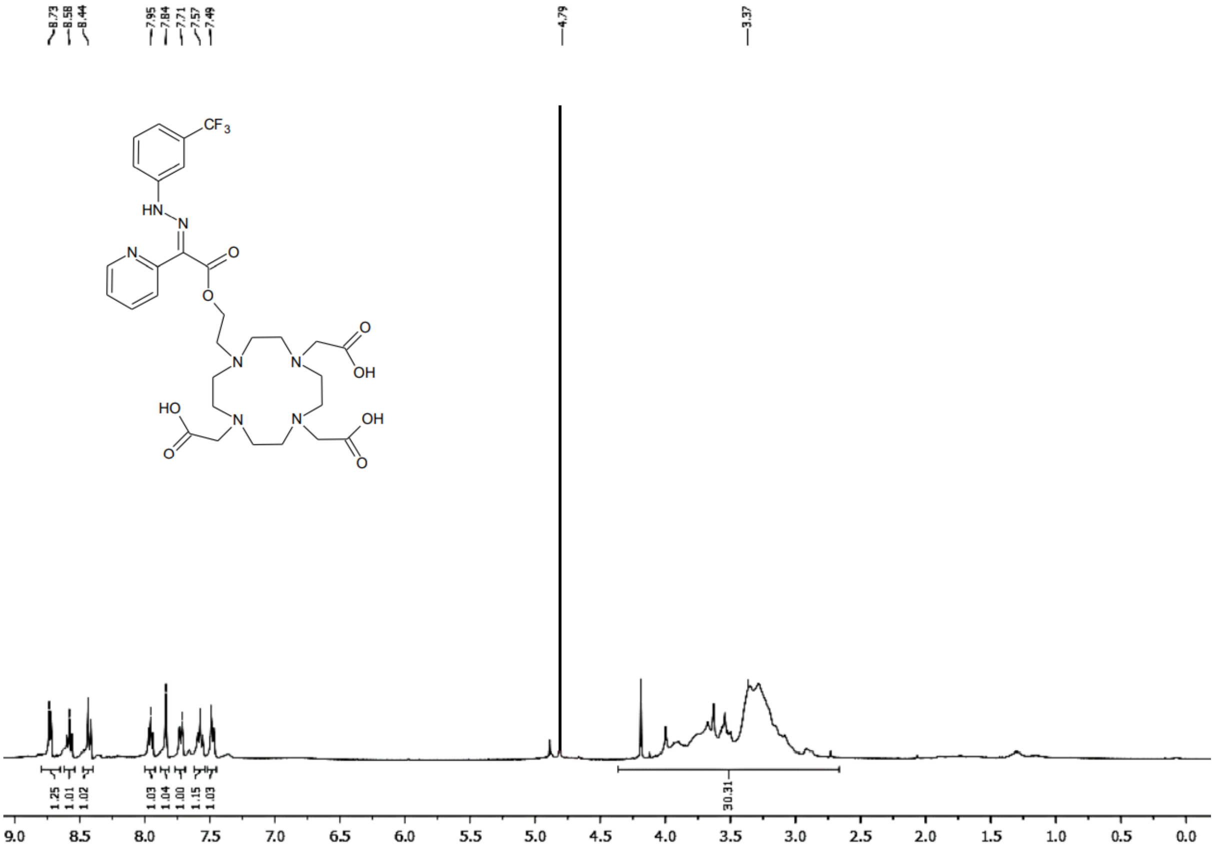

Figure S13. ^{1}H NMR spectrum of (**L**^{b}) in D_2O at 298 K.

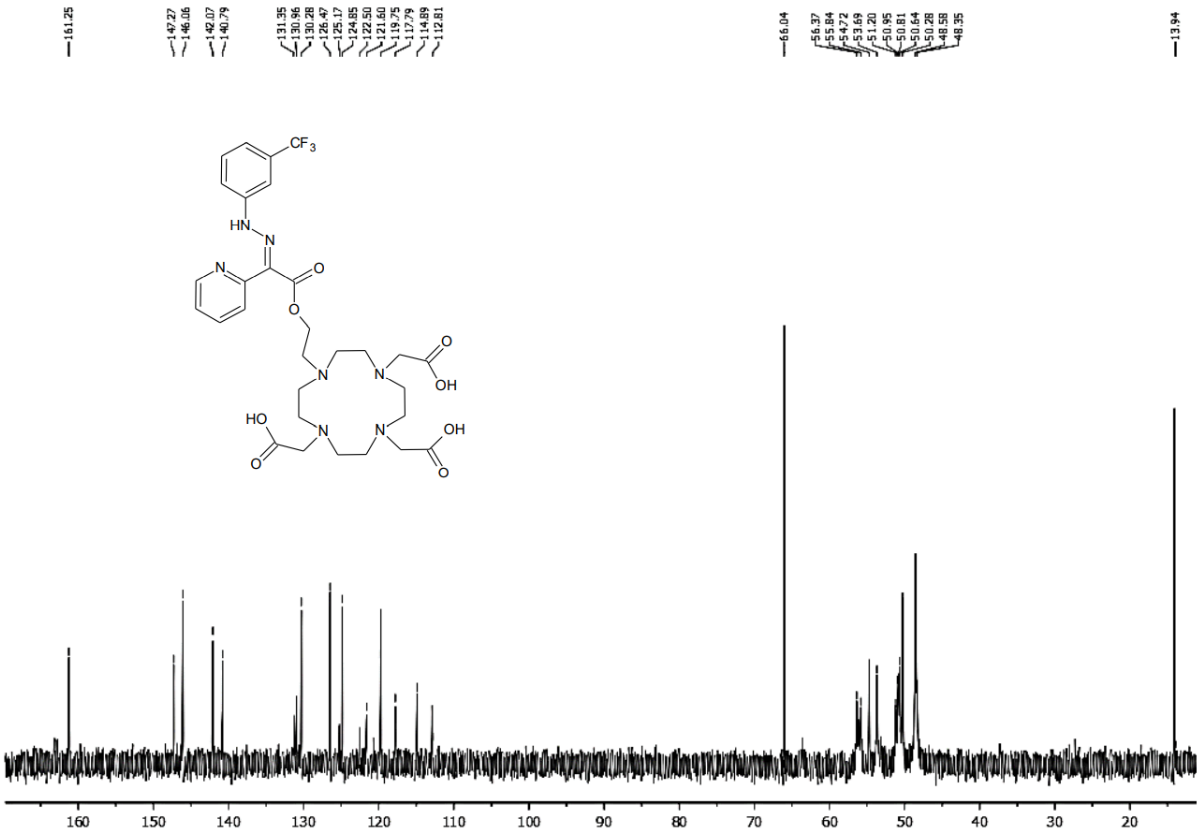

Figure S14. ^{13}C NMR spectrum of (**L**^{b}) in D_2O at 298 K.

Reversibility study

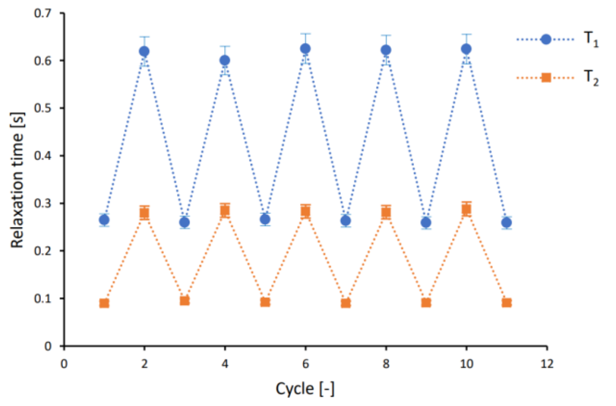

Figure S15. Relaxation times change under acidification/neutralization cycle for HoL^b solution. Blue circles – T_1; orange squares – T_2. ^{19}F NMR Agilent 400 MHz. The relaxation times of fluorine in a physiological environment such as tissues or blood at 3 T are T_1 600 ms and T_2 150 ms.[1] For our sample, we observe transitions of T_1 250-600 ms and T_2 100-300 ms, however, in pure water conditions.

^{1}H NMR relaxivity

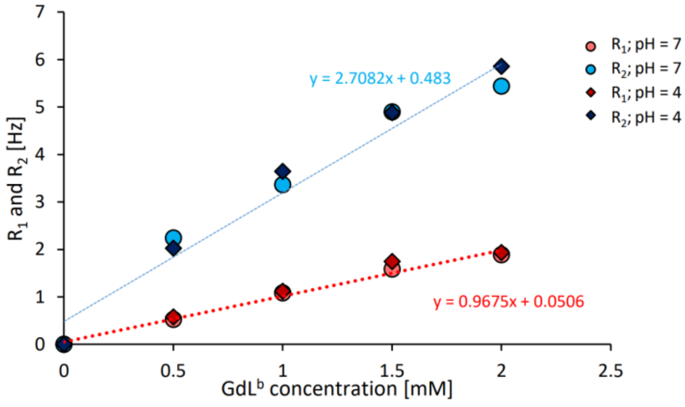

Figure S16. T_1 and T_2 relaxation times of the water protons depending on the concentration of Gd(III)L^b at pH=7 (neutral) and pH=4 (acidic).

^{19}F NMR relaxivity

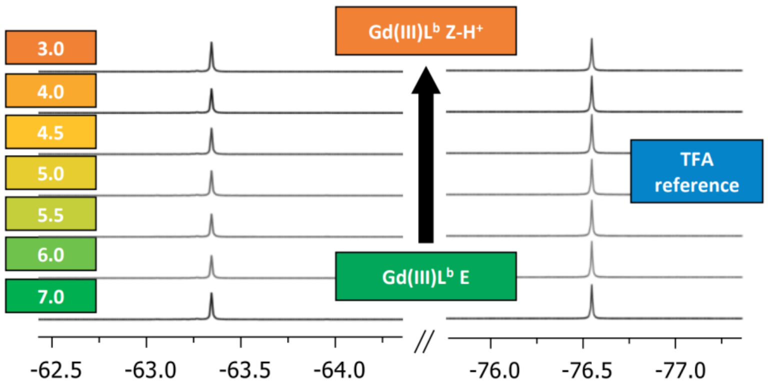

Figure S17. ^{19}F NMR signal of the CF_3 group in the Gd(III)L^b complex depending on pH. The sample was acidified with 0.01 mM HCl. Sodium salt of trifluoroacetic acid acted as a reference.

MRI

Four samples were tested, including a sample with a pH of 7 that mimics the environment of healthy tissue, a sample with a pH of 5 that represents pathological tissue, and two additional samples with pH levels of 4.5 and 4, respectively. The experiment aimed to demonstrate the switching behavior between the two pH levels of 4.5 and 4, where the latter represents the point of completed switching.

Comparison of theoretical results with experimental ^{19}F relaxations

Table S1. Calculated ^{19}F relaxation times based on Bloch-Redfield-Wangsness (BRW) theory for E and Z-H$^+$ isomers of various paramagnetic complexes of L^b. The values of T_{1e} taken from[2], assumed rotational correlation time τ_r = 0.25 ns. Relaxation time of diamagnetic reference was assumed as 1 s (T_1) and 0.5 s (T_2). Only electron-nucleus dipole-dipole and Curie interactions were included.

**Paramagnetic ion**	**Calculated relaxation times**			
	**T_1 [s]**		**T_2 [s]**	
	**9 Å**	**13 Å**	**9 Å**	**13 Å**
**Eu(III)**	0.867	0.983	0.408	0.487
**Ho(III)**	0.332	0.818	0.111	0.360
**Dy(III)**	0.324	0.813	0.110	0.360
**Gd(III)**	0.012	0.103	0.003	0.028
**Cu(II)**	0.170	0.651	0.047	0.245
**Er(III)**	0.425	0.870	0.149	0.397
**Nd(III)**	0.965	0.996	0.472	0.496
**La(III)**	1.000	1.000	0.500	0.500
**Cr(III)**	0.183	0.671	0.068	0.294
**Fe(III)**	0.156	0.627	0.056	0.269
**Co(II)**	0.291	0.788	0.111	0.361
**Yb(III)**	0.917	0.990	0.435	0.491
**Pr(III)**	0.974	0.997	0.479	0.497
**Ce(III)**	0.990	0.998	0.493	0.499

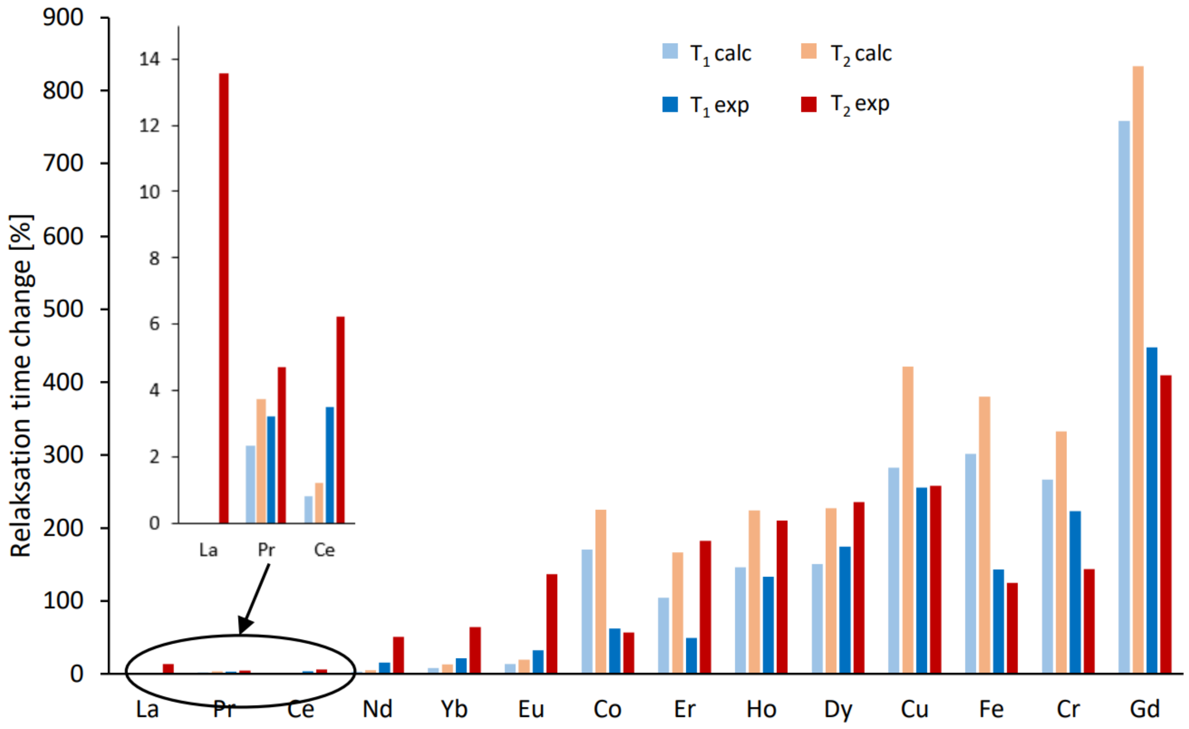

Figure S18. Relaxation time change calculated from BRW theory (T_1 calc and T_2 calc) and observed at ^{19}F NMR, 400 MHz (T_1 exp and T_2 exp) for E and Z isomers of various L^b complexes. T_{01}=1 s and T_{02}=0.5 s, B_0= 9.4 T, T= 300 K, τ_R= 0.25 ns.

BRW theoretical predictions and fitting

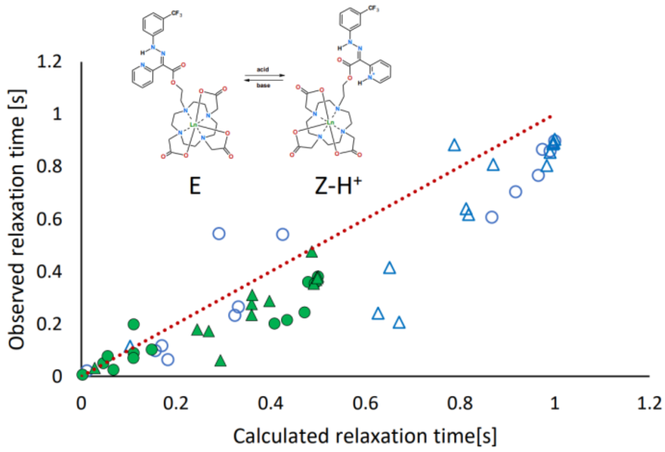

Figure S19. Comparison of ^{19}F T_1 and T_2 relaxation times. Assumptions: diamagnetic reference T_{01}=1 s and T_{02}=0.5 s, B_0= 9.4 T, T= 300 K, τ_R= 0.25 ns, distance 9.0 Å and 13.0 Å. Blue: T_1, circles (E), triangle (Z-H^+); green: T_2, circles (E), triangle (Z-H^+). The red line represents 100% accuracy.

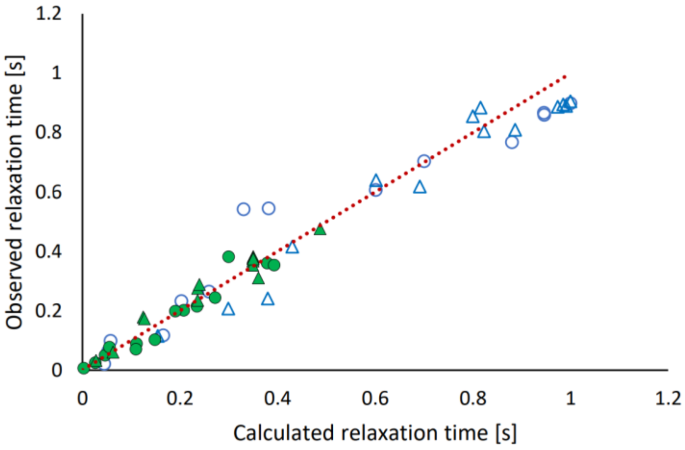

Figure S20. Comparison of ^{19}F T_1 and T_2 relaxation times. Assumptions: diamagnetic reference T_{01}=1 s and T_{02}=0.5 s, B_0= 9.4 T, T= 300 K, τ_R= 0.33 ns, distance 8.7 Å for E and 13.4 Å for Z-H^+ isomers. Blue: T_1, circles (E), triangle (Z-H^+); green: T_2, circles (E), triangle (Z-H^+). The red line represents 100% accuracy.

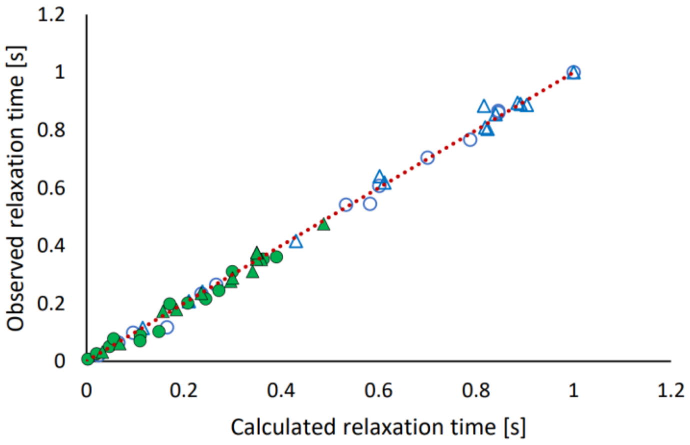

Figure S21. Comparison of ^{19}F T_1 and T_2 relaxation times. Assumptions: diamagnetic reference T_{01}=1 s and T_{02}=0.5 s, B_0= 9.4 T, T= 300 K, τ_R= 0.33 ns, calculated distances according to Table S2. Blue: T_1, circles (E), triangle (Z-H^+); green: T_2, circles (E), triangle (Z-H^+). The red line represents 100% accuracy.

Table S2. ^{19}F-M distances in L^b complexes derived from relaxation data based on BRW theory. Geometries of optimized structures showed in Table S3.

Ion	**E** [Å]	**Z** [Å]
Eu(III)	8.1	13.0
Ho(III)	9.3	13.0
Dy(III)	9.1	13.0
Gd(III)	9.0	13.0
Cu(II)	8.3	13.0
Er(III)	9.2	13.2
Nd(III)	9.0	13.1
La(III)	9.0	13.0
Cr(III)	8.3	12.8
Fe(III)	8.6	12.5
Co(II)	9.0	13.0
Yb(III)	9.0	13.0
Pr(III)	9.1	12.7
Ce(III)	9.0	12.8

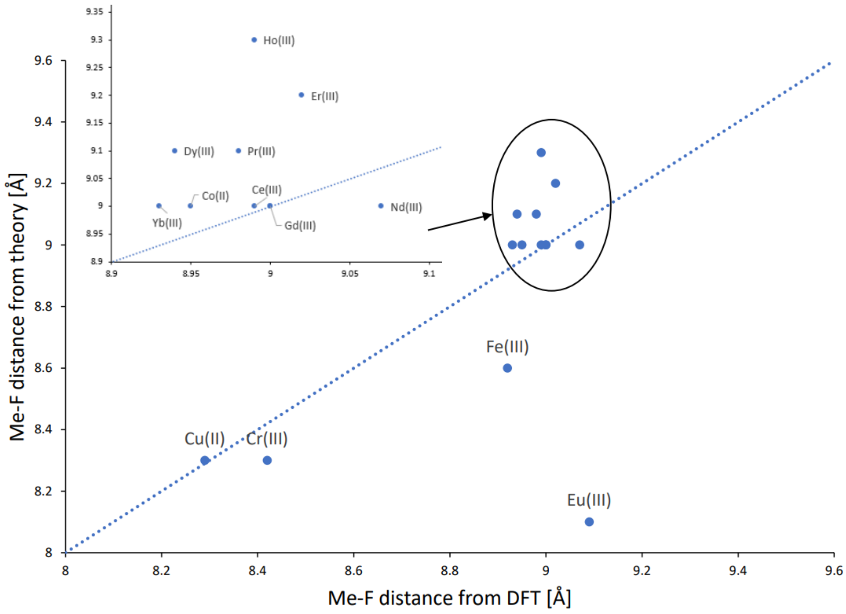

Figure S22. Comparison of the ^{19}F-M distances in L^b complexes in E form calculated by DFT (Table S2) and derived from relaxation data based on BRW theory (table S2). Assumptions: diamagnetic reference T_{01}=1 s and T_{02}=0.5 s, B_0= 9.4 T, T= 300 K, τ_R= 0.33 ns.

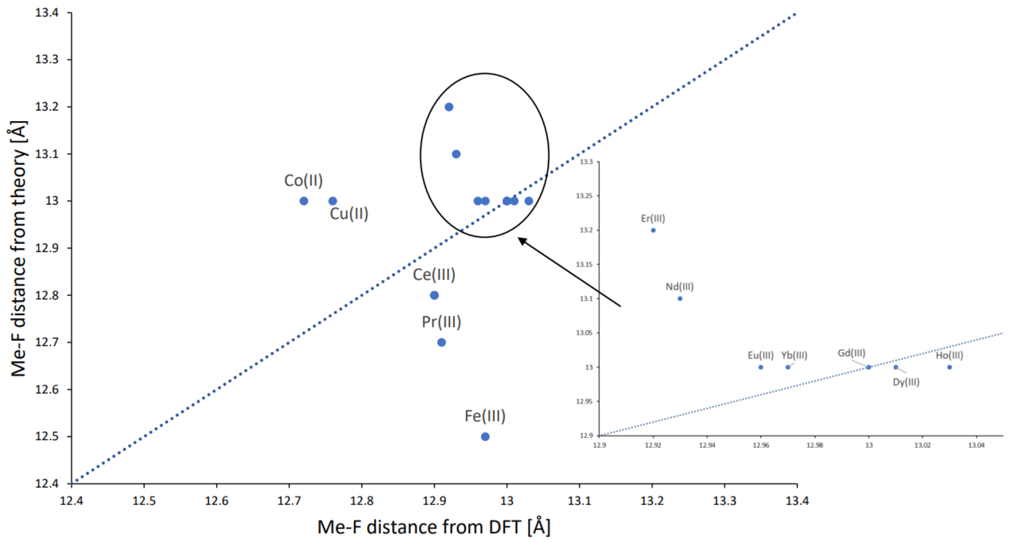

Figure S23. Comparison of the ^{19}F-M distances in L^b complexes in Z-H^+ form calculated by DFT (Table S2) and derived from relaxation data based on BRW theory (Table S2). Assumptions: diamagnetic reference T_{01}=1 s and T_{02}=0.5 s, B_0= 9.4 T, T= 300 K, τ_R= 0.33 ns.

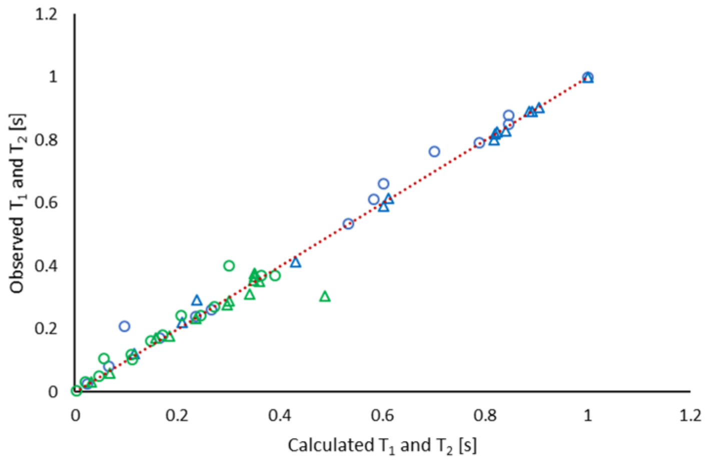

Figure S24. Comparison of observed and calculated ^{19}F T_1 and T_2 relaxation times of L^b complexes in E and Z-H^+ forms. Calculation of relaxation times based on BRW theoretical model, M-F distances from DFT for every paramagnetic ion (Table S2). Assumptions: diamagnetic reference T_{01}=1 s and T_{02}=0.5 s, B_0= 9.4 T, T= 300 K, τ_R= 0.33 ns. Blue: T_1, circles (E), triangle (Z-H^+); green: T_2, circles (E), triangle (Z-H^+).

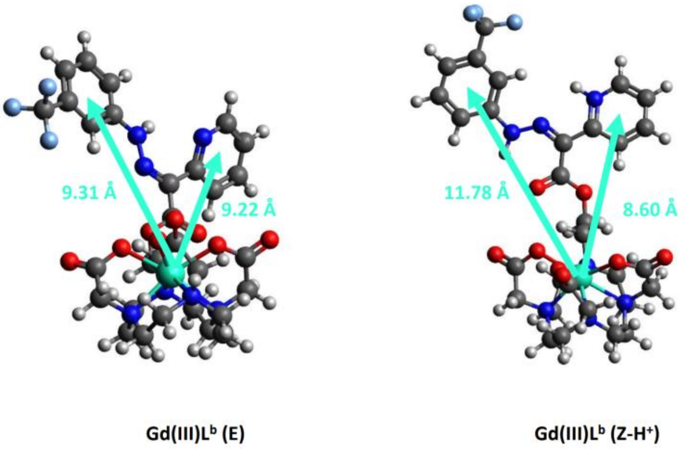

Figure S25. Distance of the metal from both aromatic rings in E and Z form.

DFT calculated structures

Table S3. Geometry optimizations of L^{a-c} complexes. All density-functional theory (DFT) calculations were performed using the Orca 4.2.1. Full geometry optimizations of L complexes were performed in aqueous solution employing hybrid metageneralized gradient approximation, with the TPSSh exchange correlation functional. In these calculations, an energy -consistent large core quasi-relativistic ECP (LCRECP) and its associated [5s4p3d]-GTO valence basis set for lanthanoid were employed, while the ligand atoms and other metals were described using the standard 6-31G(d) basis set. The input files and molecular plots were prepared with Avogadro software.

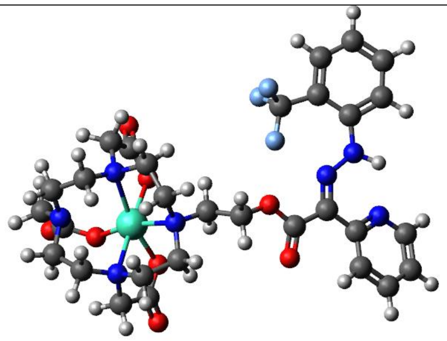

Gd(III)L^a – E

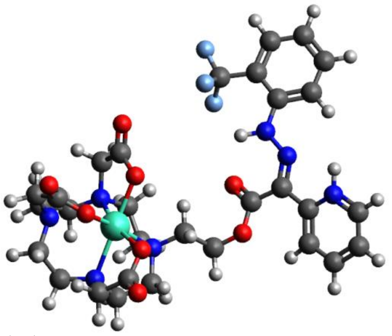

Gd(III)L^a – Z-H^+

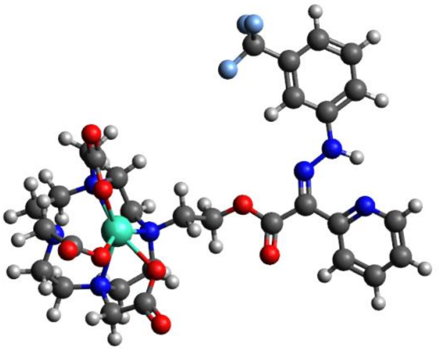

Gd(III)L^b - E

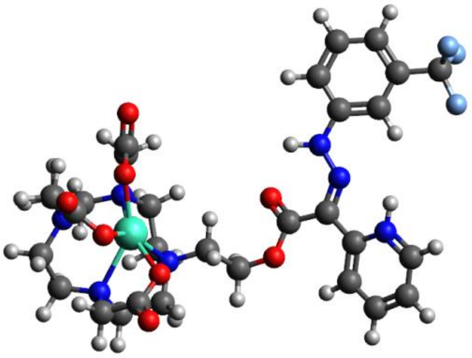

Gd(III)L^b – Z-H^+

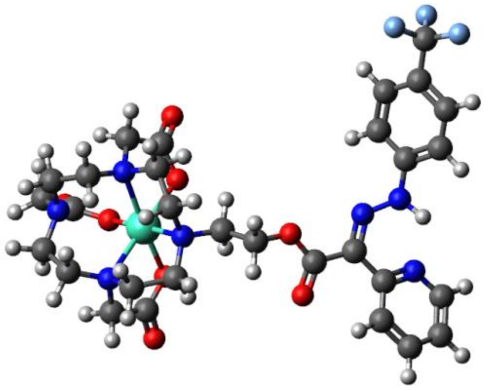

Gd(III)L^c – E

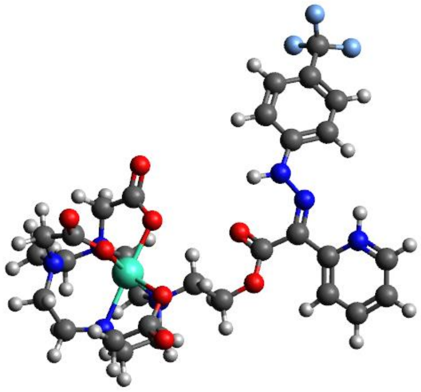

Gd(III)L^c – Z-H^+

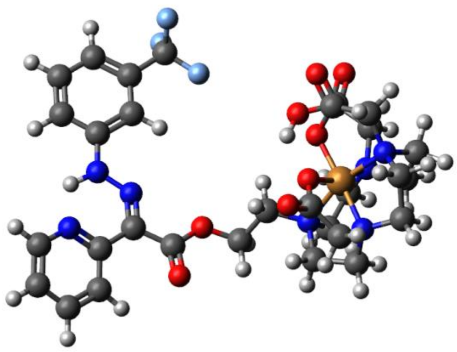

Cu(II)L^b – E (8.29 Å)

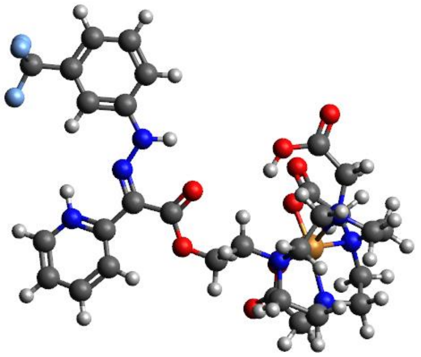

Cu(II)L^b – Z-H^+ (12.76 Å)

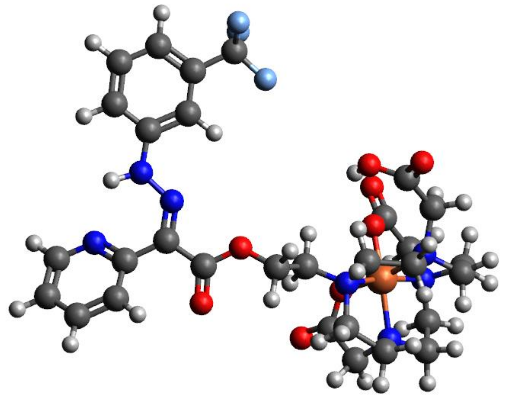

Co(II)L^b – E (8.95 Å)

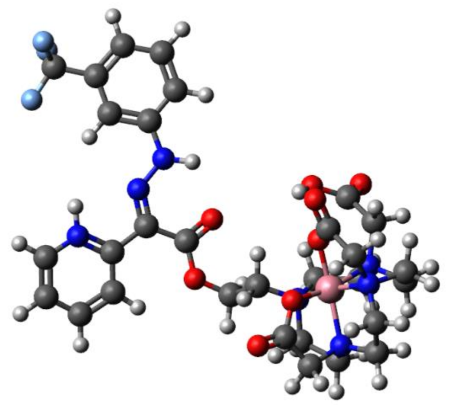

Co(II)L^b – Z-H^+ (12.72 Å)

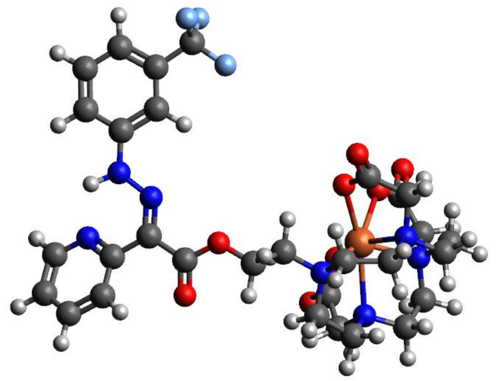

Fe(III)L^b – E (8.92 Å)

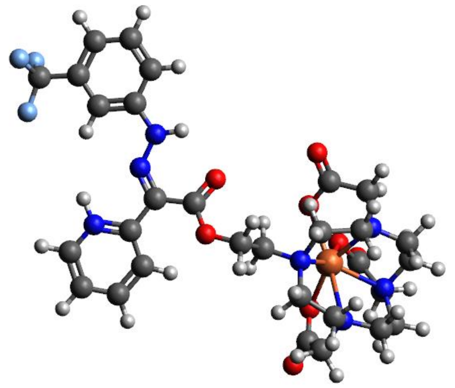

Fe(III)L^b – Z-H^+ (12.97 Å)

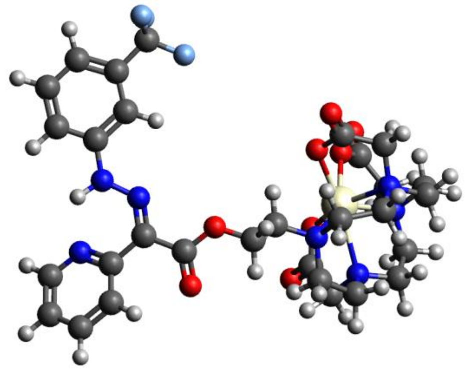

Ce(III)L^b – E (8.99 Å)

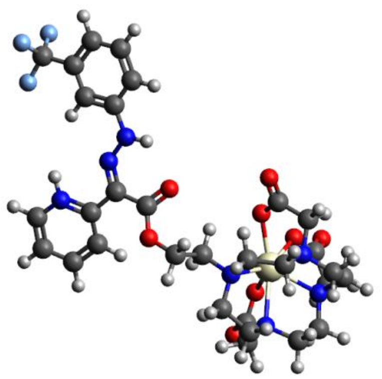

Ce(III)L^b – Z-H^+ (12.90 Å)

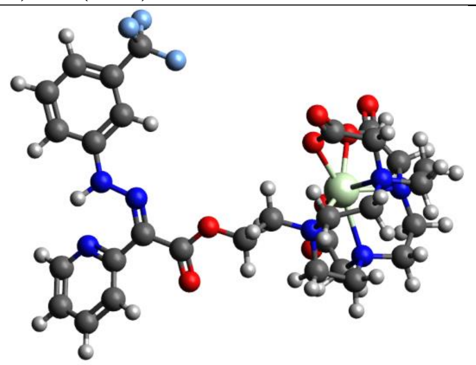

Pr(III)L^b – E (8.98 Å)

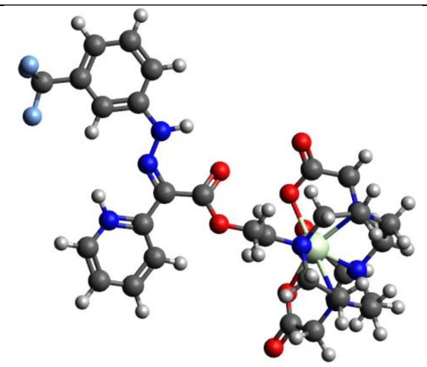

Pr(III)L^b – Z-H^+ (12.91 Å)

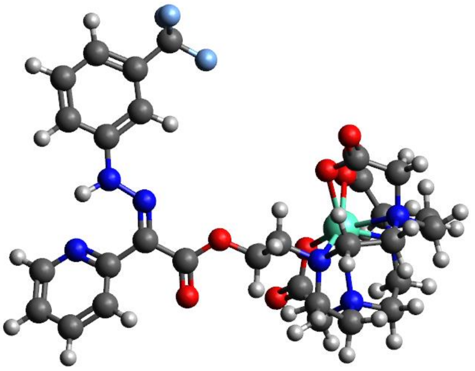

Eu(III)L^b – E (9.09 Å)

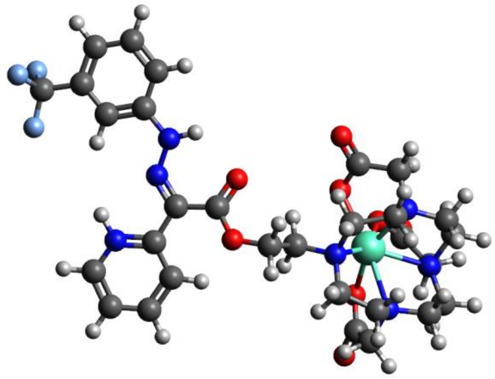

Eu(III)L^b – Z-H^+ (12.96 Å)

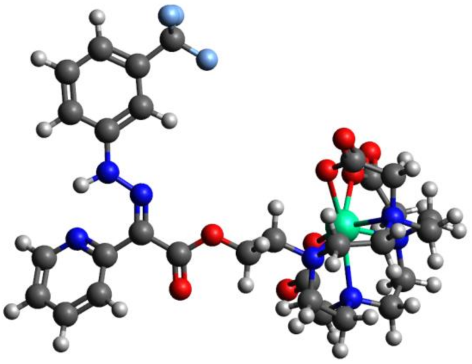

Ho(III)L^b – E (8.91 Å)

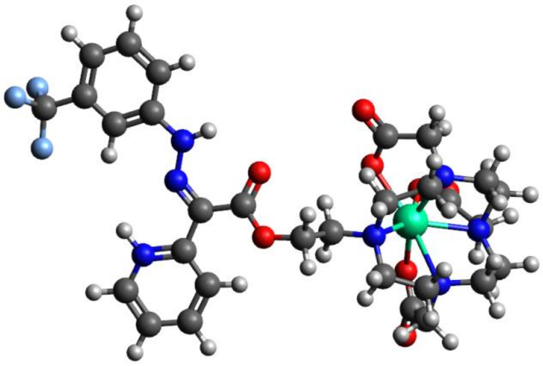

Ho(III)L^b – Z-H^+ (13.03 Å)

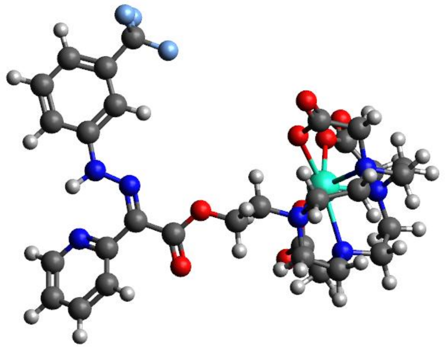

Dy(III)L^b – E (8.94 Å)

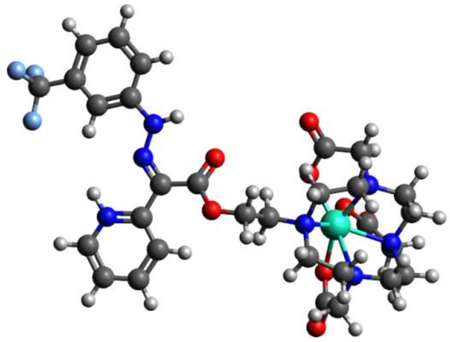

Dy(III)L^b – Z-H^+ (13.01 Å)

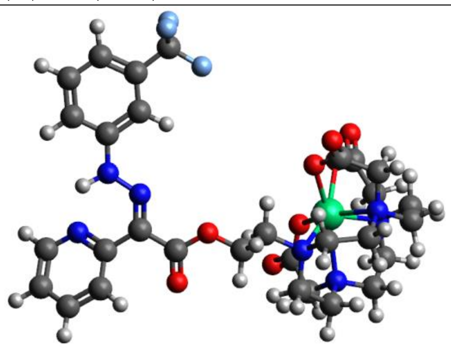

Er(III)L^b – E (9.02 Å)

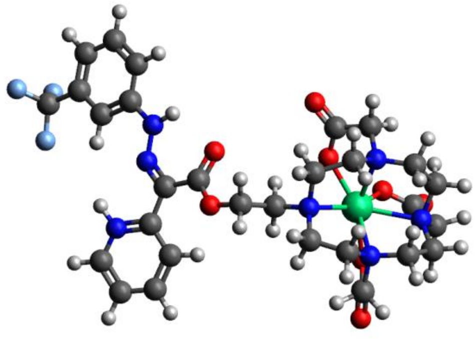

Er(III)L^b – Z-H^+ (12.92 Å)

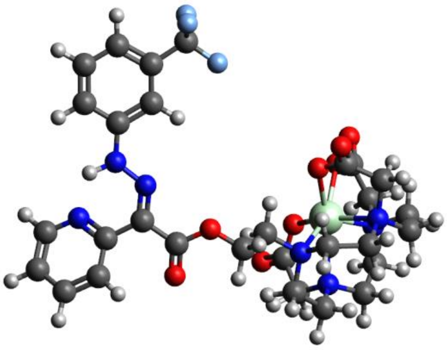

Nd(III)L^b – E (9.07 Å)

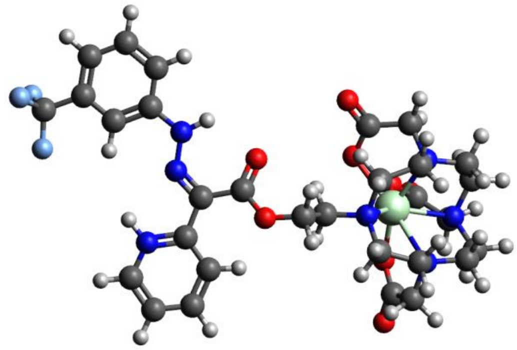

Nd(III)L^b – Z-H^+ (12.93 Å)

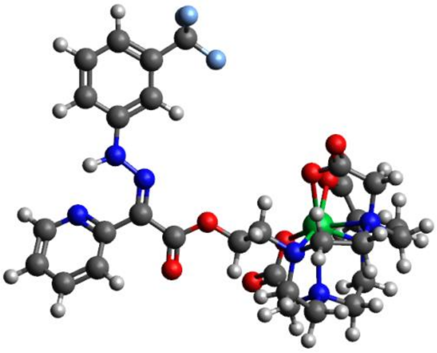

Yb(III)L^b – E (8.93 Å)

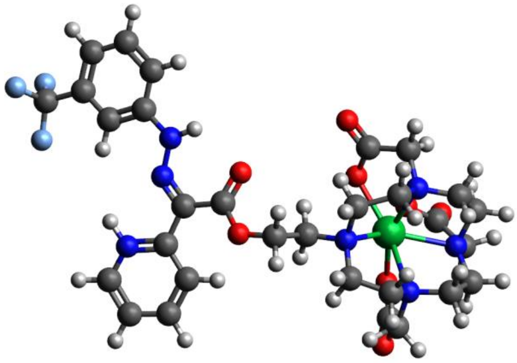

Yb(III)L^b – Z-H^+ (12.97 Å)

References

1. Colotti, R.; Bastiaansen, J.A.M.; Wilson, A.; Flögel, U.; Gonzales, C.; Schwitter, J.; Stuber, M.; van Heeswijk, R.B. Characterization of Perfluorocarbon Relaxation Times and Their Influence on the Optimization of Fluorine-19 MRI at 3 Tesla. *Magn. Reson. Med.* **2017**, *77*, 2263–2271, doi:10.1002/mrm.26317.

2. Zalewski, M.; Janasik, D.; Wierzbicka, A.; Krawczyk, T. Design Principles of Responsive Relaxometric 19 F Contrast Agents: Evaluation from the Point of View of Relaxation Theory and Experimental Data. *Inorg. Chem.* **2022**, *61*, 19524–19542, doi:10.1021/acs.inorgchem.2c03451.
